# Glucose transporter Glut1 controls diffuse invasion phenotype with perineuronal satellitosis in diffuse glioma microenvironment

**DOI:** 10.1093/noajnl/vdaa150

**Published:** 2020-10-30

**Authors:** Masafumi Miyai, Tomohiro Kanayama, Fuminori Hyodo, Takamasa Kinoshita, Takuma Ishihara, Hideshi Okada, Hiroki Suzuki, Shigeo Takashima, Zhiliang Wu, Yuichiro Hatano, Yusuke Egashira, Yukiko Enomoto, Noriyuki Nakayama, Akio Soeda, Hirohito Yano, Akihiro Hirata, Masayuki Niwa, Shigeyuki Sugie, Takashi Mori, Yoichi Maekawa, Toru Iwama, Masayuki Matsuo, Akira Hara, Hiroyuki Tomita

**Affiliations:** 1 Department of Tumor Pathology, Gifu University Graduate School of Medicine, Gifu, Japan; 2 Department of Neurosurgery, Gifu University Graduate School of Medicine, Gifu, Japan; 3 Department of Radiology, Gifu University Graduate School of Medicine, Gifu, Japan; 4 Gifu University Hospital, Innovative and Clinical Research Promotion Center, Gifu University, Gifu, Japan; 5 Department of Emergency and Disaster Medicine, Gifu University Graduate School of Medicine, Gifu, Japan; 6 Division of Genomics Research, Life Science Research Center, Gifu University, Gifu, Japan; 7 Department of Parasitology and Infectious Diseases, Gifu University Graduate School of Medicine, Gifu, Japan; 8 Division of Animal Experiment, Life Science Research Center, Gifu University, Gifu, Japan; 9 Medical Science Division, United Graduate School of Drug Discovery and Medical Information Sciences, Gifu, Japan; 10 Department of Pathology, Asahi University Hospital, Gifu, Japan; 11 Department of Veterinary Medicine, Faculty of Applied Biological Sciences, Gifu University, Gifu, Japan; 12 Center for Highly Advanced Integration of Nano and Life Sciences, Gifu University (G-CHAIN), Gifu, Japan; 13 Domain of Integrated Life Systems, Center for Highly Advanced Integration of Nano and Life Sciences (G-CHAIN), Gifu University, Gifu, Japan

**Keywords:** diffuse invasion, glioma, glycolysis, glucose transporter, perineuronal satellitosis

## Abstract

**Background:**

Gliomas typically escape surgical resection and recur due to their “diffuse invasion” phenotype, enabling them to infiltrate diffusely into the normal brain parenchyma. Over the past 80 years, studies have revealed 2 key features of the “diffuse invasion” phenotype, designated the Scherer’s secondary structure, and include perineuronal satellitosis (PS) and perivascular satellitosis (PVS). However, the mechanisms are still unknown.

**Methods:**

We established a mouse glioma cell line (IG27) by manipulating the histone H3K27M mutation, frequently harboring in diffuse intrinsic pontine gliomas, that reproduced the diffuse invasion phenotype, PS and PVS, following intracranial transplantation in the mouse brain. Further, to broadly apply the results in this mouse model to human gliomas, we analyzed data from 66 glioma patients.

**Results:**

Increased H3K27 acetylation in IG27 cells activated glucose transporter 1 (Glut1) expression and induced aerobic glycolysis and TCA cycle activation, leading to lactate, acetyl-CoA, and oncometabolite production irrespective of oxygen and glucose levels. Gain- and loss-of-function in vivo experiments demonstrated that Glut1 controls the PS of glioma cells, that is, attachment to and contact with neurons. GLUT1 is also associated with early progression in glioma patients.

**Conclusions:**

Targeting the transporter Glut1 suppresses the unique phenotype, “diffuse invasion” in the diffuse glioma mouse model. This work leads to promising therapeutic and potential useful imaging targets for anti-invasion in human gliomas widely.

**Key Points**

- Glut1-dependent glycolytic switch controls the perineuronal satellitosis, that is, attachment to and contact with neurons.
- Glut1 inhibition and metabolism-based imaging are useful for anti-invasion therapy.

**Importance of the Study**

The mechanisms controlling the diffuse invasion phenotype of gliomas are poorly understood. Recent evidence suggests that chromatin remodeling, particularly histone acetylation, is tightly linked to metabolic rewiring in tumor cells and their microenvironment. We established a glioma cell line with H3.3K27M that reproduces and recapitulates the human diffuse invasion phenotype in the mouse brain. This study makes a significant contribution to the literature because we found that Glut1-dependent metabolic rewiring via histone acetylation in glioma cells triggers conversion to the diffuse invasion phenotype. Glut1 regulation is required for glioma cells to control their invasive phenotypes via metabolic and epigenetic alterations in the glioma microenvironment. We propose a therapeutic target and potential useful imaging method, leading to an innovative anti-invasion therapy with combination of conventional antiangiogenic agents and surgical resection.

Glioma is the most common primary malignant brain tumor, accounting for approximately 30% of cases.^1^ High-grade gliomas, such as glioblastomas, are highly malignant with a median survival of 15 months.^2^ These tumors are currently incurable due to their invasive nature.^3^

This “diffuse invasion” phenotype is defined as the infiltration of glioma cells into normal brain parenchyma, which is unique to glioma cells.^4^ Glioma cells microscopically infiltrate into the space between neural cells along pre-existing blood vessels, while solid tumors in other organs show intravascular invasion and spread. Diffuse invasion without mass forming is frequently observed in diffuse intrinsic pontine glioma (DIPG).

Over the past 80 years, studies have revealed 2 key features of the “diffuse invasion” phenotype, designated the Scherer’s secondary structure, and include perivascular satellitosis (PVS) and perineuronal satellitosis (PS).^5^ These dynamic structures appear to attract glioma cells and promote their invasion^6,7^; however, the mechanisms controlling the diffuse invasion phenotype are poorly understood.

Metabolic stress, such as hypoxia and glucose deprivation, causes phenotypic switching of glioma cells.^8^ In vitro and in silico analysis suggested that aerobic glycolysis (also referred to Warburg effect) is utilized in these harsh environments.^9,10^ However, the relationship between aerobic glycolysis and the diffuse invasion phenotype remains unclear. This is due to difficulties in isolating diffuse invasive glioma cells from tissues and the lack of an easily reproducible and manageable glioma in vivo model with complete PS and PVS.^11^

Glioma cells show diverse genetic, epigenetic, metabolic, and microenvironmental compositions, leading to their phenotypic heterogeneity.^12^ A key mutation is a somatic mutation in histone *H3F3A*, leading to a lysine 27 to methionine mutation at histone H3 variant H3.3 (H3.3K27M), which is frequently identified in DIPGs.^13–15^ This H3.3K27M-mutant glioma exhibits epigenetic modifications, such as global loss of H3K27 trimethylation (H3K27me3) and reciprocal gain of H3K27 acetylation (H3K27ac).^16,17^ H3.3K27M mutation in DIPGs also enhances glycolysis and tricarboxylic acid (TCA) cycle metabolism to produce 2-Oxoglutaric acid (also referred to α-KG).^17^ However, it is unknown if these epigenetic and metabolic changes link to the diffuse invasion phenotype.

To our knowledge, an easily reproducible and manageable glioma in vivo model with complete PS is still lacking.^11,18^ Here, we established a rare glioma cell line that reproduces and recapitulates the human diffuse invasion phenotype in the mouse brain. Using this in vivo model, we investigated the mechanisms of the phenotype.

## Materials and Methods

### Ethics Statement and Study Approval

In human experiments, informed patient consent and prior approval from the Gifu University Hospital Affiliated to Gifu University Graduate School of Medicine Ethics Committees were obtained before the clinical materials were used for research purposes (no. 2018–146). All murine experiments were performed in accordance with the Gifu University International Animal Care and Use Committee guidelines for the use of animals (no. 30-21).

### Cells, Mice, and Human Samples

Immortalized glial OS3 cells (RCB1593) established from newborn C3H/HeN mice were obtained from the RIKEN Cell Bank. BALB/c nude (CAnN.Cg-Foxn1nu/CrlCrlj) mice were purchased from Charles River Japan. Human tissue sections were obtained from patients who underwent surgical resection or biopsy from 2012 to 2018 at Gifu University Hospital, Gifu, Japan.

### Cell Culture and Transfection

Cells were cultured in minimum essential medium α with GlutaMAX supplemented with 10% fetal bovine serum, 5 μg/mL bovine insulin, 0.2% D-glucose, and 0.1% penicillin-streptomycin solution at 37°C and 5% CO_2_. Cells were transfected with pcDNA3.1(+)-H3f3a-K27M plasmids and others (3.0 μg DNA per well) using Lipofectamine LTX (Invitrogen) according to the manufacturer’s instructions. Then, several clones were established by drug selection.

### Intracranial Tumor Establishment in Mice

For intracranial glioma cell implantation, 1.0 × 10^5^ cells were suspended in 1 μL phosphate-buffered saline (PBS) and stereotactically injected into the brains of 6–8-week-old male BALB/c nude at the following coordinates: 2 mm lateral and 1 mm anterior to bregma.

### Tissue Preparation

After anesthesia, the thorax of mice was opened, and an incision was made in the inferior vena cava. Perfusion washing was performed with equal volumes of cold 0.1 M PBS and cold 4% paraformaldehyde solution using a drip-infusion system. The brains were dissected, divided into pieces, and used for preparing frozen and paraffin sections.

Paraffin-embedded tissues of mouse and human were cut into 3-μm-thick serial sections, deparaffinized, and stained with hematoxylin and eosin (H&E) or used for immunohistochemistry (IHC).

The tumor area in the mouse brain that was transplanted with each cell type was measured in H&E staining sections using CellSens software (Olympus).

### Definition and Evaluation of PS and PVS

We have microscopically defined PS as follows: neurons with at least one tumor cell in contact with a neuron (Supplementary Figure S1). Five fields in each mouse were randomly selected for invasive areas of the tumor, counted at 400 × magnification, and the mean was calculated. Incidence (%) refers to the number of neurons to which glioma cells attach among the total number of neurons. PVS assessed the same way.

### Time-Lapse Imaging

The cultured cells were automatically monitored via live cell imaging system with Keyence Time Lapse Module (BZ-X800; Keyence) for up to 5 days.

### Real-Time RT-PCR

Total RNA was extracted using the RNeasy kit (Qiagen) according to the manufacturer’s protocol. cDNAs were synthesized using the SuperScript III First-Strand Synthesis Kit (Life Technologies, Inc.). Quantitative real-time RT-PCR was performed using the StepOnePlus system (Applied Biosystems). The primers that were used for real-time RT-PCR are listed in Supplementary Table S6. To analyze relative gene expression, we used the comparative Ct method. Two independent experiments were performed, with duplicate reactions in each experiment. Beta-actin was used as a housekeeping gene for normalization.

### Statistical Analysis

For mouse experiments, statistical details of experiments can be found in the figure legends. Statistical significance was determined using Mann–Whitney or Fisher’s exact tests. All statistical analyses were carried out in GraphPad Prism 6.0.

### Additional Methods

Western blotting, scanning electron microscopy, lactate assay, microarray analysis, metabolome analysis, glucose uptake assay, Seahorse XF glycolysis stress test, Chip-qPCR, and DNP-MRI imaging are described in the Supplementary Methods. All materials are listed in the Supplementary Table S1.

### Data Availability

All microarray data were deposited in Gene Expression Omnibus (GEO) under dataset accession no. GSE126725 (http://www.ncbi.nlm.nih.gov/geo/).

## Results

### Generation of Diffuse Glioma Model Induced by Gain of H3.3K27M Mutation

**Figure 1.**
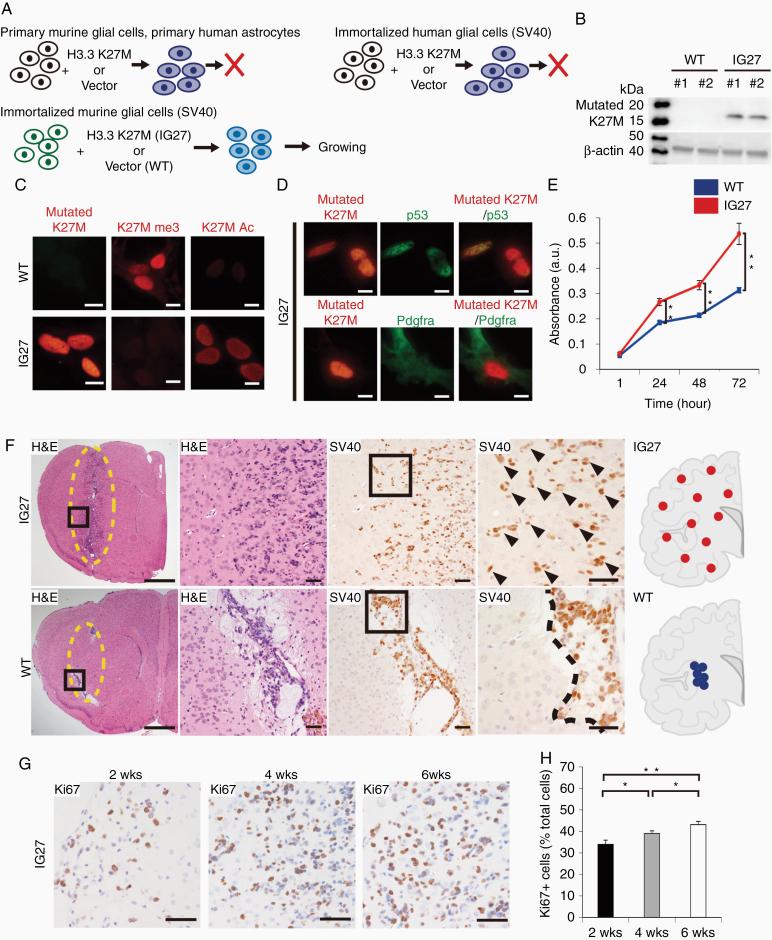
Diffuse glioma model driven by gain of H3.3K27M mutation. (A) Gene transfer via pcDNA3.1-H3.3K27M plasmid in the indicated cells. (B) Western blot analysis of IG27 and WT cells lysates with H3K27M (mutated K27M) and β-actin (*n* = 2 each) antibodies. (C) Immunocytochemistry analysis of mutated K27M, trimethyl K27me3, and K27Ac in IG27 and WT cells. Scale bars, 10 µm. (D) Double immunocytochemical localization in IG27 cells. Upper images: mutated K27M (red) and p53 (green). Lower images: mutated K27M (red) and Pdgfra (green). Scale bars, 10 µm. (E) Proliferative activity of IG27 and WT cells examined by cell counting assay. Each plot represents the mean ± SEM of 3 independent experiments. **P < .01 by Mann–Whitney test. (F) H&E staining and immunohistochemistry (IHC) for SV40 at 4 weeks following transplantation of IG27 cells (upper images) and WT cells (lower images) into mouse brains. Schematic representations of the expansion of IG27 cells (upper image, red dots) and WT cells (lower image, blue dots). Arrow heads indicate IG27 cells. Scale bars, 1 mm (leftmost images), 50 µm (right images). (G) IHC for Ki67 in mouse brains transplanted with IG27 cells. Scale bars, 50 µm. (H) Quantification of Ki67-positive cell ratio in each cohort (*n* = 2 each; 6 fields per mouse). Bars represent the mean ± SEM. *P < .05; **P < .01 by Mann–Whitney test.

We attempted to introduce an H3.3K27M plasmid vector (Supplementary Figure S2) into primary mouse glial cells isolated from neonatal mouse brains (Figure 1A); however, the transfected cells did not proliferate and stopped growing. Further, human primary astrocytes and human immortalized fetal glial cells were transfected with the H3.3K27M plasmid vector but did not proliferate. These failures are similar to those described in a previous report of immortalized normal human astrocytes.^19^

Therefore, we transfected the H3.3K27M plasmid vector into immortalized murine glial cells with SV40; these cells exhibit bipotential glial progenitor cell-like properties.^20^ We successfully generated H3.3K27M-OS3 (IG27) cells, which were able to grow, and wild type OS3 cells carrying an empty vector (WT) (Figure 1A). H3.3K27M protein expression in IG27 cells was confirmed by western blotting (Figure 1B). To evaluate histone modifications in IG27 cells, we performed IHC analysis of H3K27M, H3K27me3, and H3K27ac. IG27 cells showed increased H3K27ac expression and reduced H3K27me3 expression compared to WT cells (Figure 1C), indicating that the IG27 cells have similar histone modifications as human H3.3K27M-mutant gliomas.^16^

Further, IG27 cells showed nucleic p53 and cytoplasmic platelet-derived growth factor receptor alpha (Pdgfra) expressions (Figure 1D); this pattern is frequently observed in human H3.3K27M-mutant diffuse gliomas.^19^ The proliferative activity of IG27 cells was slightly higher than that of WT cells (Figure 1E); however, in vitro colony formation did not significantly differ between IG27 WT cells (Supplementary Figure S3).

To evaluate orthotopic tumor formation in vivo, we injected IG27 or WT cells into the midbrain in neonatal and 6–8-week-old nude mice (*n* = approximately 40 and 15, respectively). However, no tumor developed. Most of the mice died prematurely from hemorrhage and hernia. Thus, we injected into the temporal lobe of 6–8-week-old nude mice (Figure 1F). Diffuse infiltrating cells were observed in the brains injected with IG27 cells without mass formation or a necrotic core, in contrast, WT cells developed slowly as a small mass without diffuse invasion (Figure 1F).

The transplanted IG27 cells exhibited gradual, diffuse intracerebral expansion over time with high proliferation (Figure 1G and H), confirming that this model has tumorigenesis properties as in DIPG.

### H3.3K27M-Driven Glioma Model Phenocopies Diffuse Invasion of Human Gliomas

**Figure 2.**
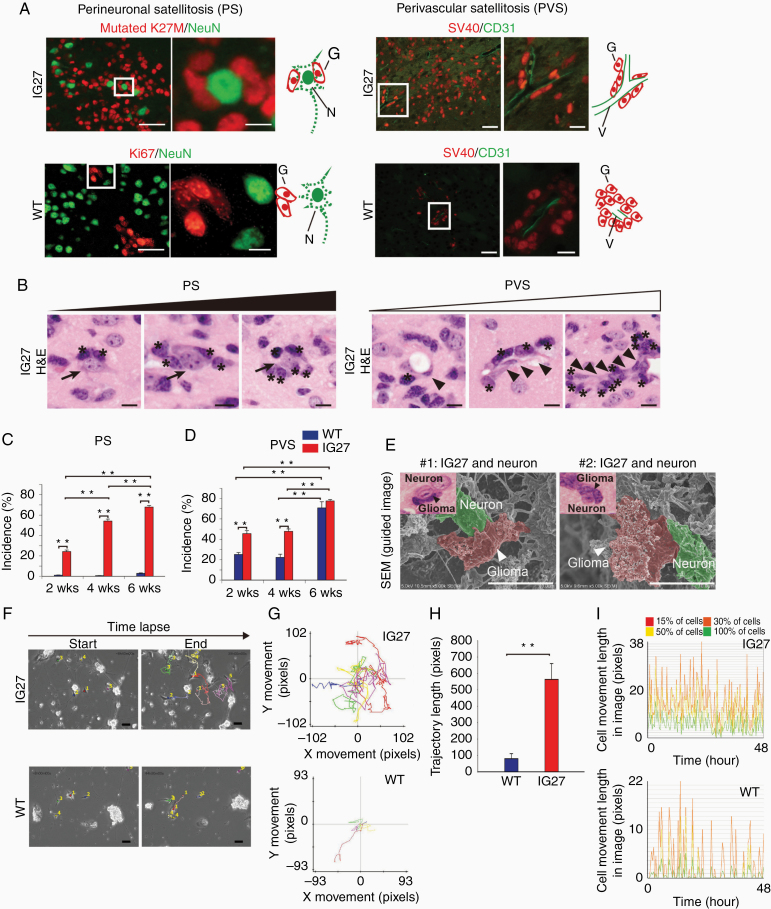
IG27-diffuse glioma reproduces diffuse invasion phenotype of human gliomas. (A) Double immunofluorescence (IF) staining of PS and PVS in IG27 cells- and WT cells-transplanted gliomas (IG27 and WT, respectively). IG27 glioma cells indicate H3-mutated K27M (red) or SV40 (red); WT glioma cells indicate Ki67 (red) or SV40 (red). Neurons indicate NeuN (green); vessels indicate CD31 (green). G, glioma cell; N. neuron; V, vessel. Scale bars, 50 µm (left images), 10 µm (right images). (B) H&E staining showing the temporal change in PS (left images) and PVS (right images), indicating the pattern by which IG27 cells accumulate and proliferate around neurons and vessels. *, IG27 cell; arrow, neuron; arrowhead, endothelial cell. Scale bars, 10 µm. (C, D) Quantitative analysis of the incidence of PS (C) and PVS (D) at 2, 4, and 6 weeks after transplantation of IG27 cells. *n* = 3 each. Bars represent the mean ± SEM. **P < .01 by Mann–Whitney test. (E) Three-dimensional (3D) images of PS by scanning electron microscopy. Scale bars, 10 µm. (F–I) Start and end images (F), centered trajectory images (G) and length (H), and cell movement length (I) of tracked IG27 (upper images) and WT (lower images) cells cocultured with primary neurons. Tracking of cells in each cohort (*n* = 5 each). Color lines indicate tracking from start to end. Bars represent the mean ± SEM. **P < .01 by Mann–Whitney test. Scale bars, 10 µm.

We assessed PS and PVS (Supplementary Figure S1), 2 key phenotypes of diffuse invasion, in IG27-diffuse glioma and control WT glioma. IG27-diffuse glioma exhibited PS and PVS with massive infiltration into the normal parenchyma (Figure 2A and B). Further, the IG27-diffuse glioma cells were also observed to infiltrate along nerve fibers in the white matter tracts (Supplementary Figure S4). In contrast, WT glioma developed much lower levels of PS than IG27-diffuse glioma (Figure 2A and B). We found that IG27 cells grew around PS and PVS (Figure 2B), mimicking the growth pattern of human malignant diffuse glioma.

To investigate the changes that occur during PS and PVS diffuse glioma progression, we analyzed the incidence of PS and PVS in IG27-diffuse glioma and control WT glioma at 2, 4, and 6 weeks after glioma cell injection into the brain. Unlike WT glioma, IG27-diffuse glioma increased the incidence of PS over time (Figure 2C). Both IG27-diffuse and WT gliomas showed an increased incidence of PVS over time (Figure 2D); however, the difference between the incidence of PVS in IG27-diffuse and WT glioma at 6 weeks was not significant (Figure 2C). These results indicate that IG27-diffuse glioma maintains the histological diffuse invasion phenotype during progression.

While the PS of Scherer’s secondary structure in human gliomas has been observed for 80 years, the actual structure of PS has been visualized only through 2-dimensional microscopes. Thus, we found that IG27 cells were rigidly attached to neurons in IG27-diffuse glioma according to 3-dimensional (3D) images obtained by scanning electron microscopy (Figure 2E).

To examine the direct relationship between IG27 cells and pre-existing neurons, we performed in vitro co-culture experiments (Figure 2F–I). Primary neurons were isolated from the brains of neonatal WT mice, cultured, and maintained to form neuron aggregates. Subsequently, IG27 or WT cells were cocultured with these neurons. Analysis by 48-h time lapse imaging and tracking of IG27 and WT cells revealed actively migrating IG27 cells around neuronal aggregates compared to WT cells (Figure 2F–I and Supplementary Movie S1–S3). The total trajectory lengths of 5 individual IG27 cells were significantly longer than 5 WT cells (Figure 2I). In sequential analyses by time lapse imaging, IG27 cells attached to and grew around neuronal aggregates (Supplementary Figure S5 and Supplementary Movie S4); however, this was uncommon for WT cells.

Collectively, 46 of 46 (100%) IG27 cell-injected mice developed diffuse gliomas, whereas 6 of 10 (60%) WT cell-injected mice developed very small gliomas with no diffuse invasion. Together, the IG27-diffuse glioma model accurately reproduces human the diffuse invasion.

### H3.3K27M-Mutant Glioma Cells Activate Cellular Glycolysis and Increase Acetyl-CoA and Oncometabolites

**Figure 3.**
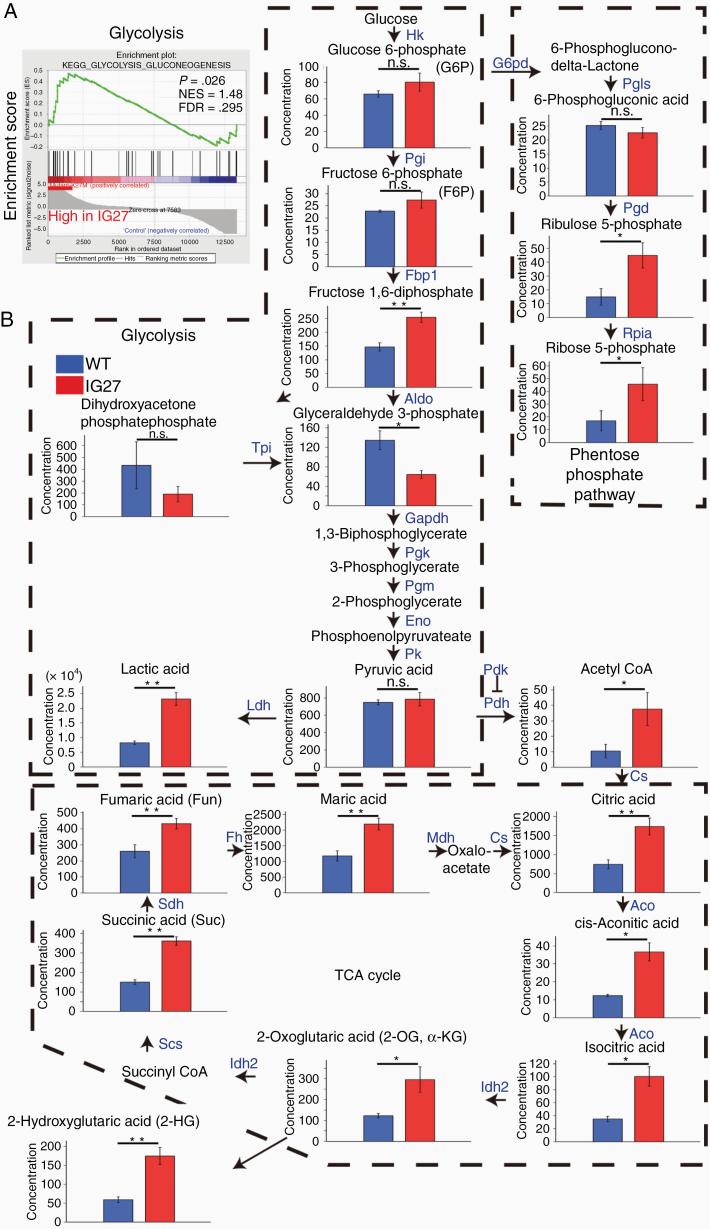
IG27 glioma cells show abundance of glycolytic metabolites and oncometabolites. (A) Enrichment plot of upregulated gene sets according to gene ontology (GO) categories (C5: GO gene sets, v6.2) in IG27 cells compared to WT cells. GSEA at cDNA microarray data from WT and IG27 cells (*n* = 3 each). NES, normal enrichment score; FDR, false discovery rate. *P*-value was calculated with weighted Kolmogorov Smirnov test. (B) Relative concentration of cellular metabolites quantified by CE-TOFMS and CE-QqQ (*n* = 3 each). Enzymes that catalyze each step are indicated in blue (see also Supplementary Tables S3 and S4). Concentration indicates pmol/1 × 10^6^ cells. Bars represent the mean ± SD. *P < .05, ** *P* < .01 by Welch *t*-test.

Next, we performed transcriptional profiling and gene set enrichment analysis^21^ for IG27 and WT cells. Gene ontology analysis revealed 303 gene sets significantly enriched in IG27 compared to WT cells (*P* < .001;Supplementary Table S2). Particularly, genes associated with glycolysis were associated with the molecular phenotypes of IG27 cells (Figure 3A), which showed significantly increased levels of stemness-related genes^22^ (Supplementary Figure S6), indicating the stem cell-like properties of these cells.

To investigate the cellular metabolic status of IG27 cells, we conducted metabolome analysis to quantify 116 target metabolites by capillary electrophoresis mass spectrometry in IG27 and WT cells. Profiling showed that many metabolites of the glycolysis pathway, PPP, and TCA cycle were increased in IG27 cells compared to WT cells (Figure 3B, Supplementary Tables S3 and S4).

Interestingly, IG27 cells showed accumulation of oncometabolites, that is, 2-HG, succinate, and fumarate (Figure 3B), which are related to carcinogenesis via HIF stabilization and epigenetic modifications.^23^

Acetyl-CoA is a central metabolite in histone acetylation and closely associated with glioma progression with metabolic reprogramming; increases in its levels trigger site-specific H3K27ac regulation, leading to upregulation of cell adhesion genes.^24^ Acetyl-CoA was markedly increased in IG27 cells compared to WT cells (Figure 3B), suggesting that acetyl-CoA is associated with maintaining H3K27 acetylation in diffuse glioma cells.

### H3.3K27M-Mutant Glioma Cells Maintain Glucose Uptake and Lactate Secretion Independently of Harsh Extracellular Conditions

**Figure 4.**
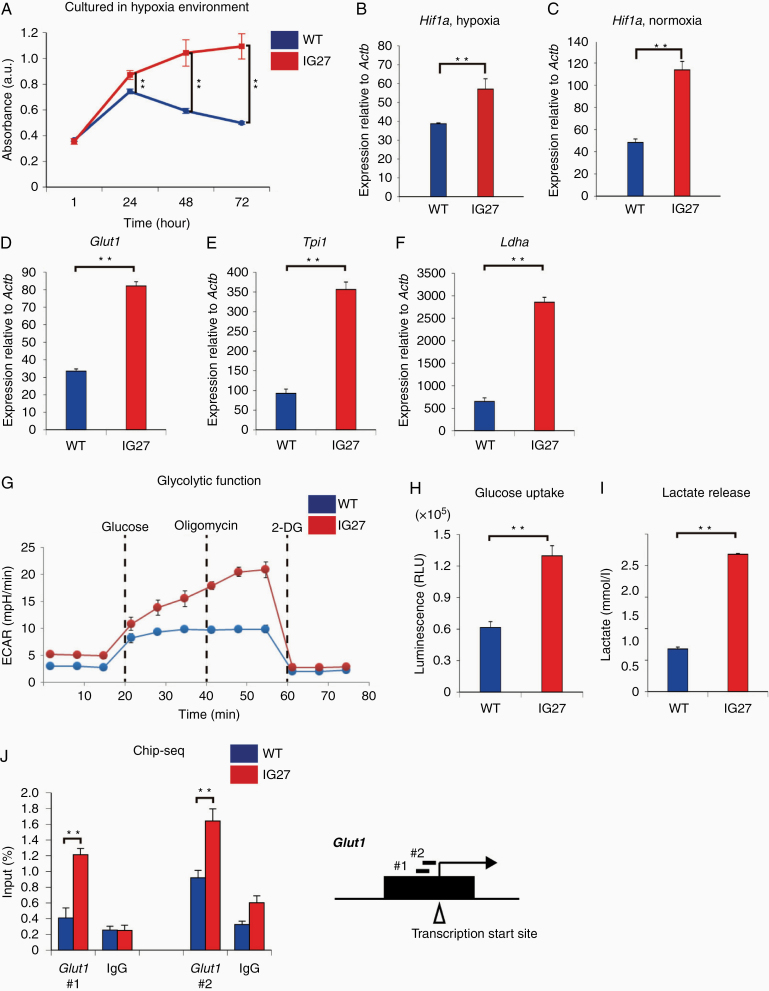
IG27 glioma cells enhance glycolysis independently of oxygen level. (A) Proliferative activity of WT and IG27 cells examined by cell counting assay under hypoxia (O_2_, 3%). Each plot represents the mean ± SEM of 3 independent experiments. **P < .01 by Mann–Whitney test. (B, C) Relative *Hif1a* RNA expression levels detected by real-time RT-PCR under low (O_2_, 3%) (B) and normal (C) O_2_ levels in WT and IG27 cells. (D–F) Relative RNA expression levels of *Glut1* (D), *Tpi1* (E), and *Ldha* (F) detected by real-time RT-PCR in WT and IG27 cells. (G) Glycolysis stress test using Seahorse XFp analyzer (*n* = 3 each, each plot indicates the mean ± SD). (H, I) Glucose uptake (H) and lactate production (I) in WT and IG27 cells. (J) ChIP-qPCR analysis using control IgG and histone H3K27ac antibody in WT and IG27 cells. The ChIPs were subjected to qPCR using oligonucleotides to amplify the promoter regions of *Glut1* (*n* = 3 each). (B–F, H–J) Bars represents the mean ± SEM of 3 independent experiments. **P < .01 by Mann–Whitney test.

Cancer cells undergo metabolic reprogramming to adapt to stress conditions related to the extracellular environmental composition, including hypoxia and glucose deprivation, for their survival.^25,26^ So far, IG27 cells alter their intracellular levels of gene expression and metabolite related to glycolysis, gliomagenesis, and stemness (Figures 3 and 4). Thus, we next focused on the extracellular condition of IG27 cells.

To determine whether the proliferation of IG27 cells is altered under hypoxia conditions, we cultured and compared IG27 and WT cells under low oxygen levels (hypoxic, 3% O_2_). Cultured IG27 cells, but not WT cells, continued to proliferate under low oxygen levels for 72 h (Figure 4A). *Hif1a* RNA expression was significantly higher in IG27 cells than in WT cells under hypoxic and normoxic conditions (Figure 4B and C). This suggests that high *Hif1a* RNA expression, without relying on oxygen levels, allows IG27 cells to grow.

The glucose transporter Glut1 takes up glucose from the extracellular space (derived from the bloodstream) into cells, and HIF1 upregulates GLUT1 to increase glucose uptake into cells and upregulates lactate dehydrogenase A (LDHA) in the termination of glycolysis.^27^ Thus, we confirmed the high RNA expression of *Glut1*, *Tpi-1*, and *Ldha*, in IG27 and WT cells under normoxic conditions (Figure 4D–F).

Next, to investigate real-time glycolysis-metabolic activities in IG27 cells under normoxic conditions, we performed a glycolysis stress test using a Seahorse XF Analyzer in IG27 and WT cells (Figure 4G). IG27 cells showed an increased extracellular acidification rate compared to WT cells (Figure 4G), demonstrating that glycolysis, glycolytic capacity, and glycolytic reserve were increased in IG27 cells. Further, both glucose uptake and lactate release were increased in IG27 cells (Figure 4H and I).

### Increased H3K27ac Restores Glut1 Expression in H3.3K27M-Driven Glioma Cells

IG27 glioma cells showed increased H3K27ac (Figure 1C). Recently, H3K27ac global deposition was shown to alter gene expression in H3.3K27M-mutant diffuse glioma.^16,28^ Thus, to determine if the increase in H3K27ac directly alters *Glut1* expression, we performed ChIP-qPCR using an H3K27ac antibody in IG27 and WT cells (Figure 4J). The result revealed a significant increase in H3K27ac-associated lesions around the transcriptional start site of *Glut1* (Figure 4J). Histone deacetylase treatment decreased the Glut1 transcription although there was not significant (Supplementary Figure S7). These results suggest that IG27 cells enhance their glycolytic metabolism and *Glut1* transcriptional activation via increased H3K27ac.

### Glut1 Controls the PS of Glioma Cells, Leading to Diffuse Invasion

**Figure 5.**
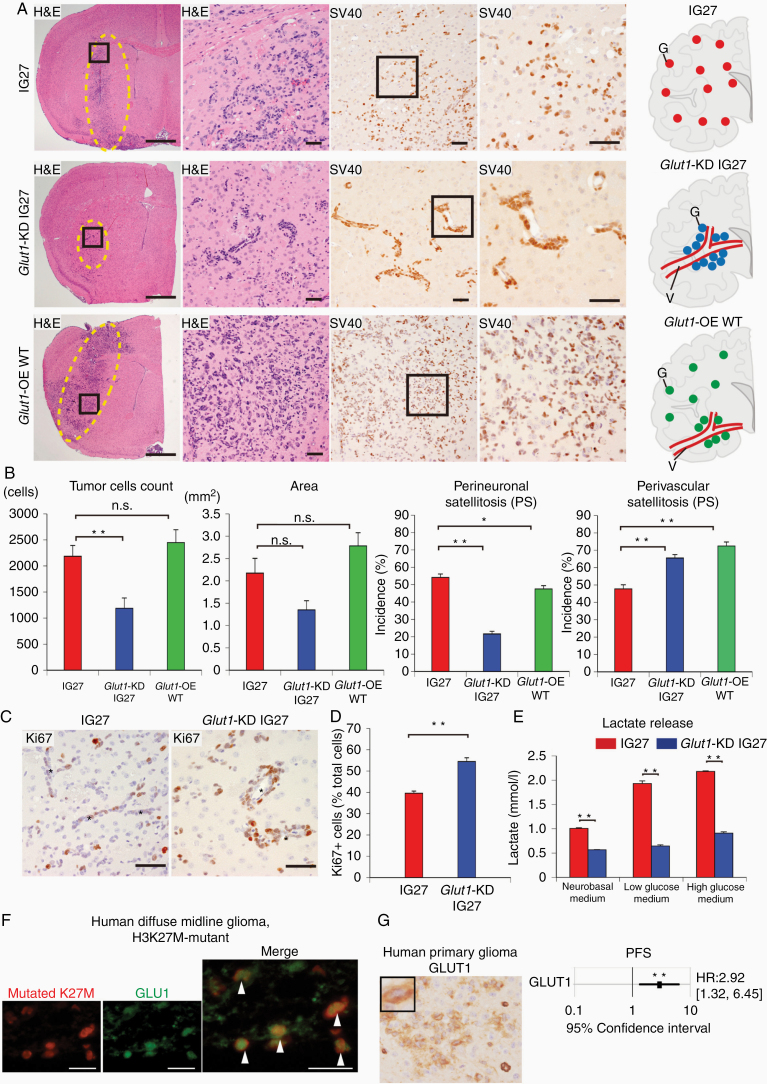
Glut1 controls the PS of glioma cells, leading to diffuse infiltration. (A) Representative images of IG27-glioma, *Glut1*-KD IG27 glioma, and Glut1-OE WT glioma. H&E staining and IHC for SV40 at 4 weeks after transplantation of IG27 cells (upper images), *Glut1*-KD IG27 cells (middle images), and Glut1-OE WT cells into mouse brains. Schematic distributions of IG27 cells (upper, red dots), *Glut1*-KD IG27 cells (middle, blue dots), and *Glut1*-OE WT cells (lower, green dots) in mouse brains. V, vessel. Scale bars, 1 mm (leftmost images), 50 µm (right images). (B) Quantitative analysis of tumor cell number, tumor expansion area, incidence of PS and PVS at 4 weeks after transplantation of IG27 glioma cells, Glut1-KD glioma, and Glut1-OE WT glioma (*n* = 6 each in 5 fields per mouse). Incidence, number of neurons to which glioma cells attached among the total number of neurons. (C) IHC for Ki67 in IG27 glioma and *Glut1*-KD IG27 glioma. Asterisk indicates vessel. Scale bars, 50 µm. (D) Quantification of Ki67-positive cell ratio in each cohort (*n* = 3 each; 6 fields per mouse). (E) Lactate levels in cell culture supernatants of IG27 and *Glut1*-KD IG27 cells in the indicated media for 24 h. Data were obtained from 3 independent experiments. (F) Double IF staining for histone H3 mutated K27M (red) and GLUT1 (green) in H3K27M-mutant diffuse glioma of human patient’s tissue. Arrowheads, double positive glioma cells. Scale bars, 50 µm. (G) (left) Representative IHC image of GLUT1 in the infiltrative area of human primary gliomas within the normal brain parenchyma. An inset showed GLUT1 membranous expression. Scale bars, 50 µm. (right) Multivariate analysis adjusted for age, recurrence, sex, and histological classification for progression-free survival. **P < .01. Statistical test is described in Methods. HR, hazard ratio. (B, D, E) Bars represent the mean ± SEM. n.s., not significant; *P < .05; **P < .01 by Mann–Whitney test.

To identify genes controlling histological “diffuse invasion” phenotypes in IG27-diffuse glioma in vivo, we generated IG27 cells with stably suppressed *Hif1a*, *Ldha*, *Tpi1*, and *Glut1* expression using short hairpin RNA (Supplementary Figure S8). The 2 clones that were knocked down most strongly were used in each gene. These cells were injected into mouse brains, and tumor cell number, tumor expansion area, and the incidence of PS and PVS were assessed (Figure 5A and B and Supplementary Figure S9).

Among these tumors, *Glut*1- and *Hif1a-*knockdown (KD) IG27 gliomas showed significantly reduced tumor expansion areas and cell numbers compared to control IG27-diffuse glioma (Figure 5A and B and Supplementary Figure S9). *Tpi1*-KD IG27 glioma cells failed to successfully engraft, and *Ldha*-KD IG27 glioma exhibited an increased tumor expansion area and cell number (Supplementary Figure S9). Although *Glut*1- and *Hif1a-*KD IG27 gliomas showed significantly reduced PS and increased PVS, with *Glut1*-KD IG27 glioma exhibiting PVS but not PS clearly (Figure 5A and B).

Next, we performed gain of function experiments of Glut1 in WT glial cells. We transfected a Glut1-overexpressing (OE) vector into WT glial cells to generate stable Glut1-OE WT cells (Supplementary Figure S10). Following injection of the cells into the mouse brains, Glut1-OE WT cells became diffuse glioma with diffuse invasion phenotypes (both PS and PVS) (Figure 5A and B).

To compare the importance of Glut1 and Hif1a in the diffuse invasion phenotype, we also generated Hif1a-OE WT cells (Supplementary Figure S11); however, Hif1a-OE WT cells did not reproduce this phenotype (both PS and PVS) in the brain.

Together, Glut1, but not Hif1, controls the PS of glioma cells, which may allow glioma cells to infiltrate the surrounding normal brain parenchyma in our H3.3K27M- driven diffuse glioma in vivo model.

### Glut1 Regulates Stable Lactate Release in Diffuse Invasion of IG27 Glioma Cells

To determine if Glut1 affects tumor proliferation in the perivascular niche, we analyzed the proliferative activity of IG27 and *Glut1-*KD IG27 glioma cells (Figure 5C). The Ki67-positive cell ratio of Glut1-KD IG 27 glioma cells in the perivascular space was significantly higher than that in control IG27-glioma (Figure 5D), suggesting that this theory partially depends on Glut1 regulation.

A heterogeneous acidic environment and hypoxia conditions are fundamental for inducing glioma invasion.^29^ Thus, we analyzed lactate release levels in the supernatant of *Glut1*-KD IG27 and control IG27 glioma cells in high-(3 g/L) and low-glucose (1 g/L) glial cell medium and neuronal growth medium. The results revealed significantly reduced lactate production in *Glut1*-KD IG27 glioma cells compared to control IG27 cells in all media (Figure 5E), suggesting that Glut1 is crucial for maintaining lactate release under the harsh nutrients conditions in the brain.

### GLUT1 Expression in Diffuse Invasion Is Associated With Early Progression in Human Glioma Patients

To determine whether GLUT1 is associated with H3.3K27M in human, we performed double immunofluorescent (IF) staining for H3.3K27M and GLUT1 in human H3.3K27M-mutant diffuse glioma tissues. The results revealed coexpression of nuclear H3.3K27M and membranous GLUT1 in H3.3K27M-mutant glioma cells but not endothelial cells (Figure 5F and G and Supplementary Figure S12).

To evaluate whether our in vivo results widely extend to various types of gliomas with diffuse invasion, we collected samples from 66 patients with confirmed gliomas including nearly all subtypes of gliomas (Supplementary Table S5), and then performed IHC for GLUT1 (Figure 5G). There were 44 (66.7%) positive cases of GLUT1 when GLUT1 membranous expression was evaluated in the invasive front of diffuse infiltrative glioma cells independent of necrotic areas (Figure 5G and Supplementary Table S5). The predictive prognostic value of these proteins was analyzed by Cox proportional hazard regression models. Univariate analysis revealed no significant differences in progression-free survival and overall survival time associated with GLUT1 expression (Figure 5G, Supplementary Figure S13). However, multivariable analysis of progression-free survival revealed that patients with GLUT1-positive cells have a 2.92-fold higher risk of progression than those with GLUT1-negative cells (95% CI: 1.32–6.45, *P* = .008, Figure 5G). The results of multivariable analysis of overall survival were not significant (Supplementary Figure S13).

### Selective Glut1 Inhibitor Suppresses Invasion of H3.3K27M-Mutant Diffuse Glioma Model

**Figure 6.**
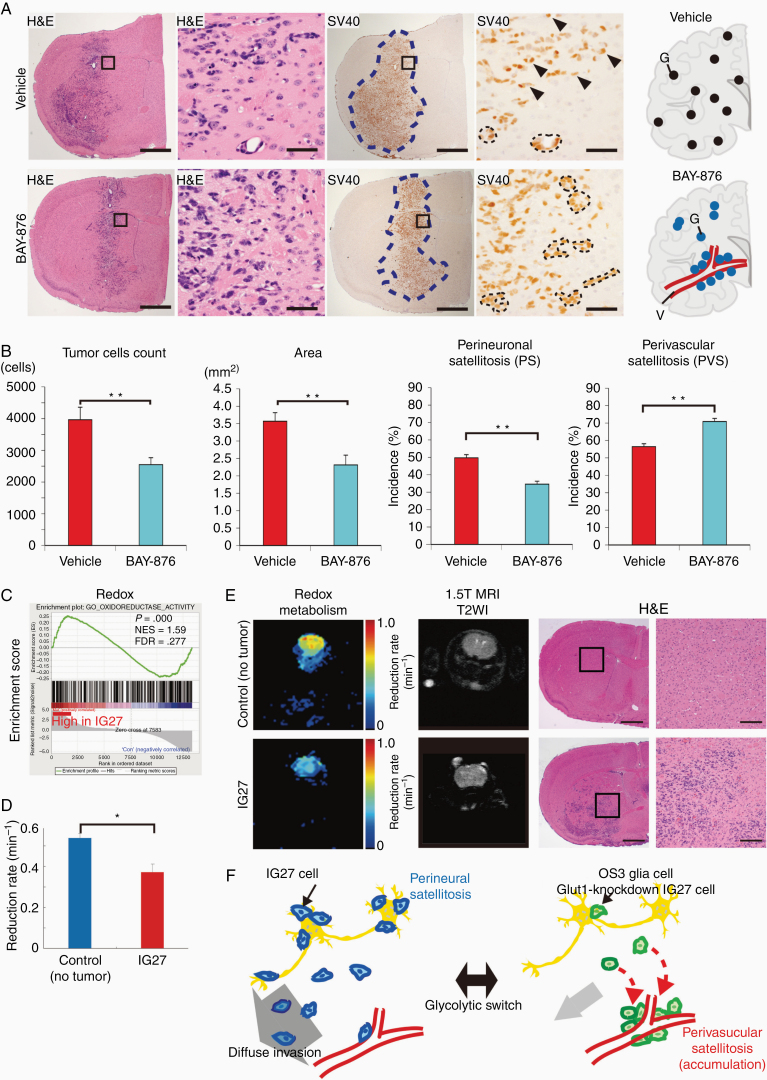
Glut1 inhibitor is effective as anti-invasive therapy and metabolism-based imaging is useful for delineating invasion area. (A) Representative H&E staining and IHC for SV40 in IG27 gliomas of nontreated (vehicle) and BAY-876- treated mice. Schematic distributions of IG27 glioma cells (upper, black dots) in brains of nontreated mice, IG27 glioma cells (lower, blue dots) in brains of BAY-876-treated mice. Scale bars, 1 mm (low powers), 50 µm (high power images). Arrowhead, IG27 cell; region among dotted lines, PVS. (B) Quantitative analysis of tumor cell number, tumor expansion area, incidence of PS and PVS in IG27 gliomas of nontreated (vehicle), and BAY-876-treated mice (*n* = 8 and 9, respectively). Incidence, number of neurons to which glioma cells attach among the total number of neurons. Bars represent the mean ± SEM. **P < .01 by Mann–Whitney test. (C) Enrichment plot of redox reaction gene sets in IG27 cells compared to WT cells. GSEA at cDNA microarray data from WT and IG27 cells (*n* = 3 each). *P* < .001, false discovery rate <25%. (D) Time-course DNP-MR images of the head region in control (no-cell transplanted) and IG27-transplanted mice after injection of MC-P. Graph shows the reduction rate in the whole brain region in each group. Bars represent the mean ± SD; *P < .05; *n* = 4–5. (E) Comparison of redox metabolic map (DNP-MRI), anatomical images (MRI), and pathological images (H&E). Redox metabolic maps (left images) of control (no tumor) (upper images) and IG27-transplanted mice (lower images). Redox metabolism maps were calculated using time lapse DNP-MR images. 1.5-T MR images were obtained after in vivo DNP-MR imaging (center images). H&E images show the pathological distribution of IG27 cells (right images). Scale bars, 1 mm (H&E, left), 200 µm (H&E, right). (F) Schematic diagram of diffuse invasion with PS controlled by the glycolytic switch in IG27 and WT glia cells (or Glut1-Kockdown IG27 cells) in the brain microenvironment. WT, wild type.

To evaluate whether Glut1 inhibitors suppress invasion, we administrated selective Glut1 inhibitors, WZB117 and BAY-876, to the mice bearing IG27-diffuse glioma. Following administration, all mice were healthy without any side effects. Whereas WZB117 treatment did not suppress the tumor cell number or expansion area (Supplementary Figure S14), BAY-876 treatment significantly reduced the tumor cell number, expansion area, and PS incidence compared to that in the nontreated control group (Figure 6A and B). In contrast, the PVS incidence in the BAY-876-treated group was significantly higher than in nontreated IG27 glioma (Figure 6B), which is similar to the progression and invasion phenotype of *Glut1*-KD IG27 glioma (Figure 5A and B). These results suggest that the selective Glut1 inhibitor BAY-876 suppresses diffuse invasion phenotype with PS.

### Metabolic Alteration-Based Imaging Is Useful for Delineating Diffuse Infiltrating Area of Glioma

MRI is widely used to characterize gliomas; however, it remains difficult to detect the diffuse infiltration into the normal brain parenchyma.^30^ Recently, Magnetic resonance spectroscopy (MRS) imaging can assess metabolic alterations in H3K27M-mutated glioma cell lines and human tissues.^17^ Our group has studied dynamic nuclear polarization (DNP)-MRI and MRS, which enables early detection of abnormal metabolism and monitoring disease progression, compare with MRI by specific probes.^31^ Thus, we conducted dynamic nuclear polarization (DNP)-MRI with a contrast agent, methoxycarbonyl PROXYL (MC-P), in IG27-diffuse glioma. Because this imaging can detect redox metabolism in vivo, we predicted its usefulness for delineating IG27 cells with the redox metabolism gene signature (Figure 6C).

Pharmacokinetic images obtained by DNP-MRI every 30 s after injection of MC-P solution (150 mM isotonic solution, 7.5 μL/g of body weight) showed that the image intensity in the mouse head region was immediately increased (Supplementary Figure S15). The MC-P (oxidized form) signal intensity reached a maximum at 30 s after injection and decreased during the subsequent 5 min. The DNP signal intensity decayed significantly more slowly (Figure 6D), in mice transplanted with IG27 cells than in mice that were not transplanted with cells. A previous study demonstrated that tumor tissue aggregates show a faster reduction rate because of the high reactive oxygen species production, hypoxic conditions, and high antioxidant levels within the tumor.^32^

Redox metabolism in brain tissue invaded by IG27 cells showed a lower MC-P reduction rate throughout the brain without tumor (Figure 6D). In the redox metabolism imaging of Figure 6E, the blue color showed a lower MC-P reduction rate in brain tissue invaded by IG27 cells. On the other hand, the yellow color showed a normal MC-P reduction rate in brain tissue. The tumor invasion area can be identified by this contrast, although T2-weighted image (T2WI) revealed no contrast change following IG27 cell infiltration (Figure 6E).

## Discussion

In our established diffuse glioma model, Glut1 deregulation was essential for the PS of glioma cells and subsequently for preferential invasion with PS (Figure 6F).

We focused on the histopathological invasive phenotype, particularly PS, affected by BAY-876 treatment. Preclinical studies have focused on the use of transporters involved in glycolysis as anticancer targets by small molecules.^33^ Among the reported GLUT inhibitors, BAY-876 is the only inhibitor that is both highly potent and selective for GLUT1 over other glucose transporters, including WZB-117.^34^ Thus, it is likely that the results show a difference in selectivity to Glut1 in our study. While it is still unclear to evaluate a survival benefit to BAY-876 in this study, scaled-up pharmacological studies, including those to evaluate other Glut1 inhibitors, are needed. Although BAY-876-treated mice showed no side effect and were healthy until the endpoint, side effects must be carefully examined because Glut1 is highly expressed in the endothelial cells of the blood–brain barrier.^35^

DNP-MRI can detect various metabolic alteration signals depending on the probes’ sensitivity. Interestingly, the brain with IG27-diffuse glioma showed a lower metabolic signal than the brain of mice not transplanted with cells. One reduction site of MC-P is the electron transport chain in the mitochondrial inner membrane^31^; therefore, IG27-diffuse glioma infiltration into the brain tissue may decrease the reduction rate by reducing electron transport chain activity in the mitochondria of tumor cells. The mechanism determined by DNP-MRI should be assessed in further preclinical studies.

While there is a limitation of SV40 in the IG27 glioma model, we believe its rarity and importance as a diffuse glioma mouse model is beyond its limitation. Actually, SV40 is an unnatural mechanism through which to engineered replicative immortality and lacks direct relevance to human glioma pathobiology. However, the IG27 glioma model certainly recapitulates a diffuse invasion with Scherer’s secondary structures as human diffuse gliomas show histopathologically with stem cell-related gene signature of human gliomas. Our results suggest that although they exhibit slight deviation from human H3K27M-gliomas, IG27 is useful for studying the glioma microenvironment and cellular mechanisms of glioma cells.

Recently, it has been reported that the immortalized mouse neuronal stem cells that express H3.3K27M was generated as a DIPG model, and concluded that H3.3K27M mutation in DIPGs enhances glycolysis and TCA cycle metabolism.^17^ The results of decreased H3K27me3, increased H3K27ac, and increased 2HG in that study are closely similar to our results on epigenetic and metabolic status. Our study shows that, increased H3K27ac caused by a gain of H3K27M regulates glycolytic metabolism via Glut1 regulation in PS of glioma diffuse invasion. However, to determine if elevated levels of 2HG would be correlated with H3K27M, further studies are needed. On the other hand, injection of our IG27 cells into the midline and brainstem of neonatal mice failed to reproduce tumors. Studying our IG27 cells may provide further insight into the mechanisms of DIPG.

We propose a therapeutic target and potential useful imaging method for evaluating glioma invasion, leading to a novel anti-invasion therapy with combination of antiangiogenic drugs and surgical resection. Although intra- and extratumoral heterogeneity dynamically and rapidly changes in the glioma microenvironment, the epigenome-metabolism-invasion axis in its microenvironment is an important clue to develop novel therapies and imaging methods for gliomas.

## Supplementary Material

vdaa150_suppl_Supplementary_Materials

vdaa150_suppl_Supplementary_Movie-S1

vdaa150_suppl_Supplementary_Movie-S2

vdaa150_suppl_Supplementary_Movie-S3

vdaa150_suppl_Supplementary_Movie-S4
