# Supplementary material for: Glucose transporter Glut1 controls diffuse invasion phenotype with perineuronal satellitosis in diffuse glioma microenvironment
Source: Neurooncol Adv. 2020 Oct 30;3(1):vdaa150. doi: 10.1093/noajnl/vdaa150 (PMC7817894; doi:10.1093/noajnl/vdaa150)
Supplement: vdaa150_suppl_Supplementary_Materials [file vdaa150_suppl_supplementary_materials.docx]

**Supplementary information**

**Supplementary Figure S1-S15**

**Supplementary Methods**

**Supplementary Table S1-S6**

**Glucose transporter Glut1 controls diffuse invasion phenotype with perineuronal satellitosis in diffuse glioma microenvironment**

Masafumi Miyai, Tomohiro Kanayama, Fuminori Hyodo, Takamasa Kinoshita, Takuma Ishihara, Hideshi Okada, Hiroki Suzuki, Shigeo Takashima, Zhiliang Wu, Yuichiro Hatano, Yusuke Egashira, Yukiko Enomoto, Noriyuki Nakayama, Akio Soeda, Hirohito Yano, Akihiro Hirata, Masayuki Niwa, Shigeyuki Sugie, Takashi Mori, Yoichi Maekawa, Toru Iwama, Masayuki Matsuo, Akira Hara, and Hiroyuki Tomita ^*^

**Supplementary Figure S1**

**
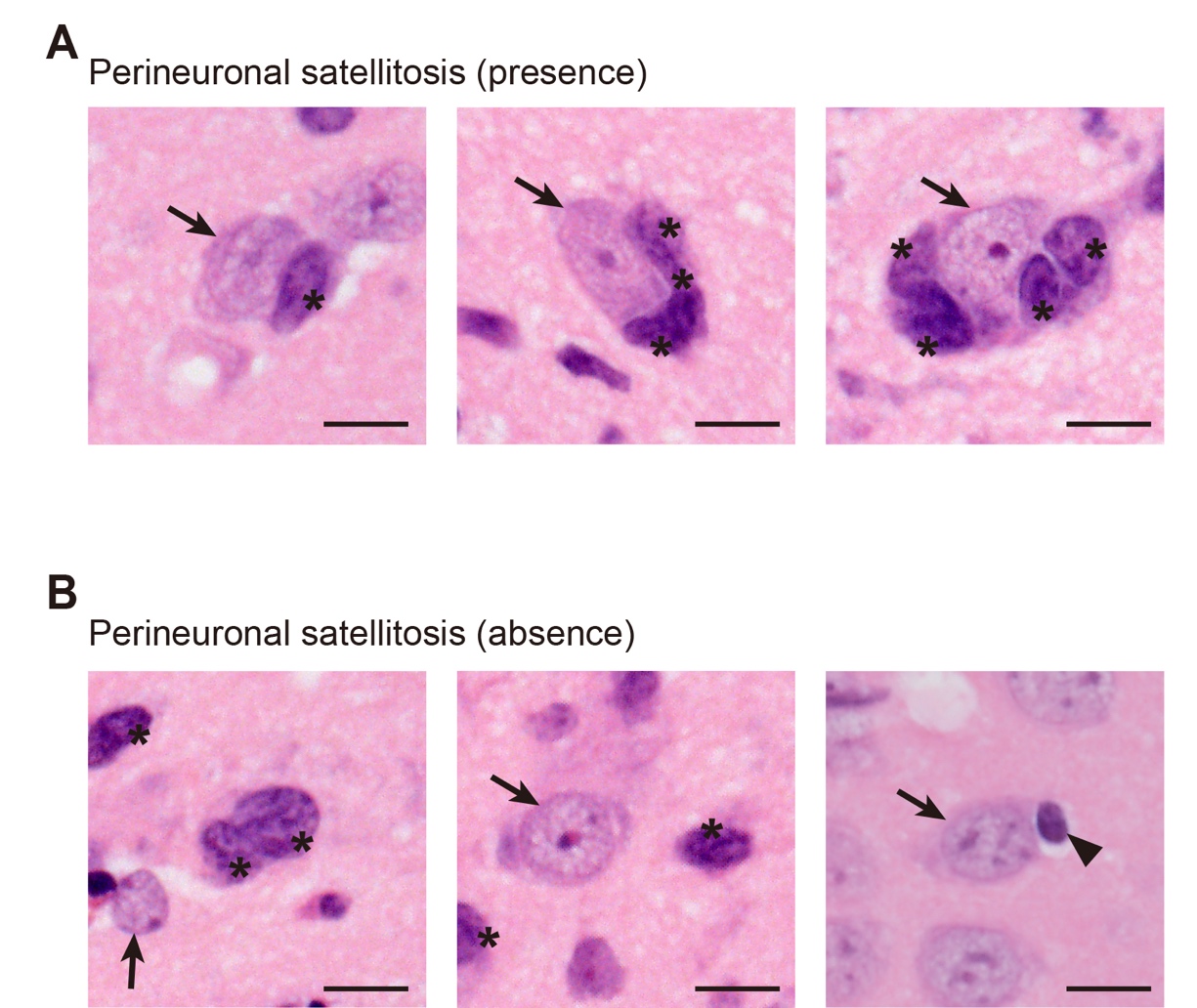
**

**Supplementary figure S1:** **Definition of perineuronal satellitosis (PS).**

**(A)** Representative H&E staining of PS-presence.

**(B)** Representative H&E staining of PS-absence.

Arrows, neurons. *, glioma cells. Arrowheads, glial cells. Scale bars, 10 µm.

**Supplementary Figure S2**


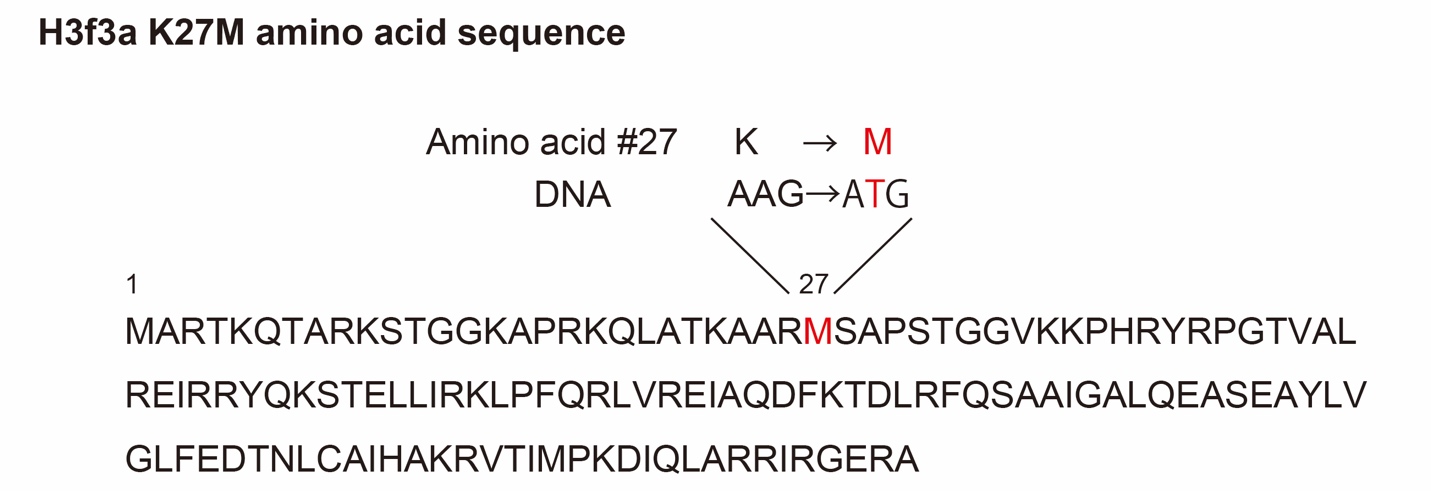


**Supplementary figure S2:** **Histone H3.3K27M mutation amino acid sequences.**

H3.3K27M amino acid sequence used in this study.

**Supplementary Figure S3**

**
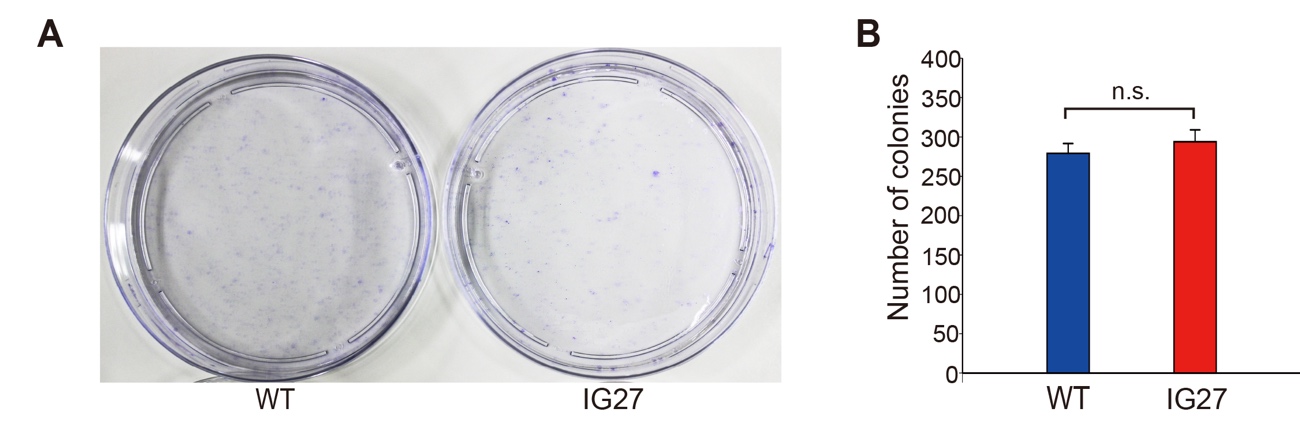
**

**Supplementary figure S3:** **Analysis of colony formation assay.**

**(A)** Representative images of colony forming assay in IG27 and WT cells.

**(B)** Number of colonies in IG27 and WT cells of triple independent experiments. Bars represent mean ± SD. n.s., not significant.

**Supplementary Figure S4**


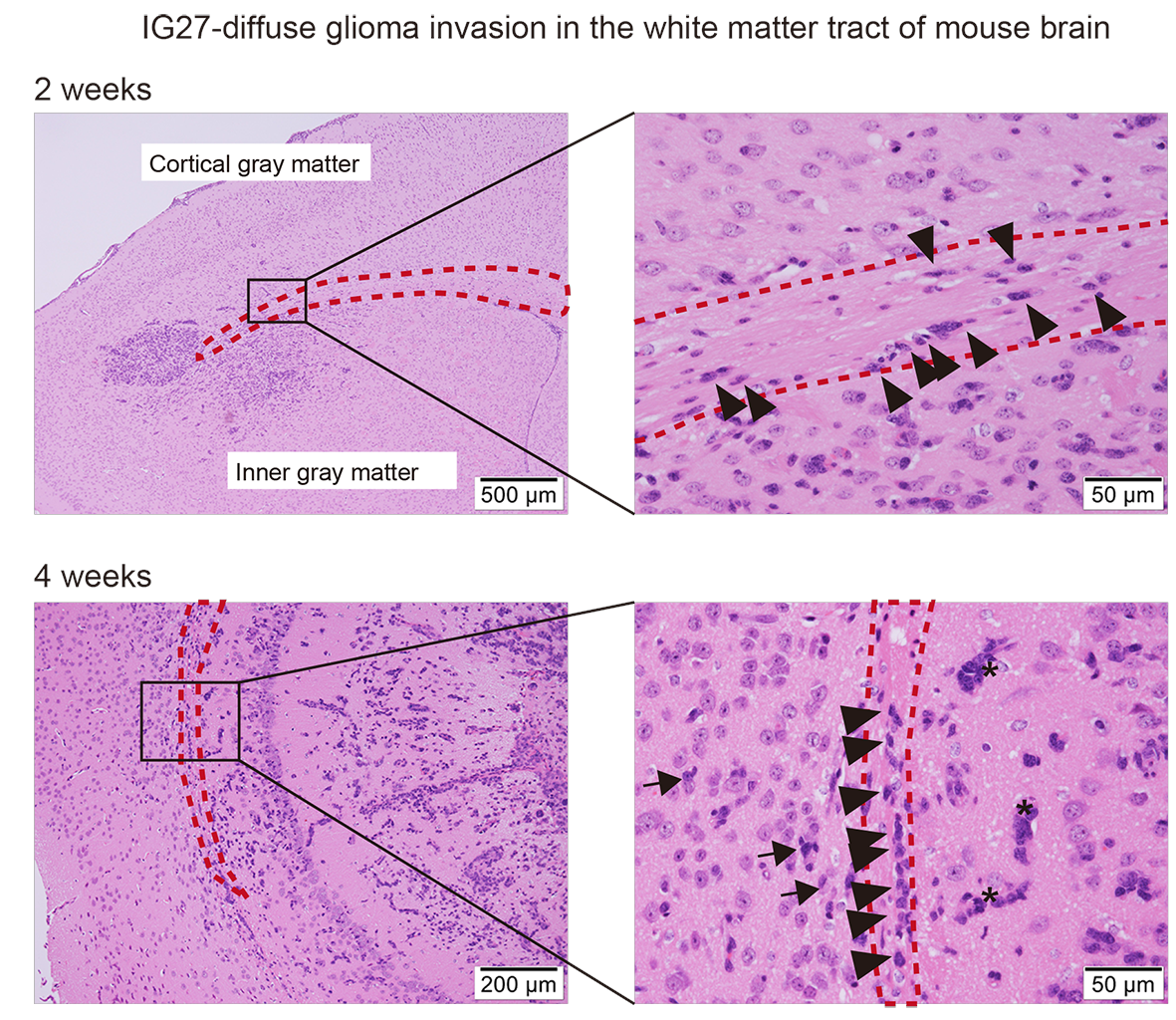


**Supplementary figure S4: The diffuse infiltration of IG27-glioma cells along the white matter tracts.**

Representative H&E photos of IG27-diffuse gliomas along nerve fibers in the white matter tracts in mouse brains at 2 and 4 weeks after the IG27 cell-transplantation. Perineuronal satellitosis (PS) and perivascular satellitosis (PVS) are also observed. Red dot lines surround the white matter tracts in mouse brain. Arrowheads, glioma cells; Arrows, PS; asterisks, PVS.

**Supplementary Figure S5**

**
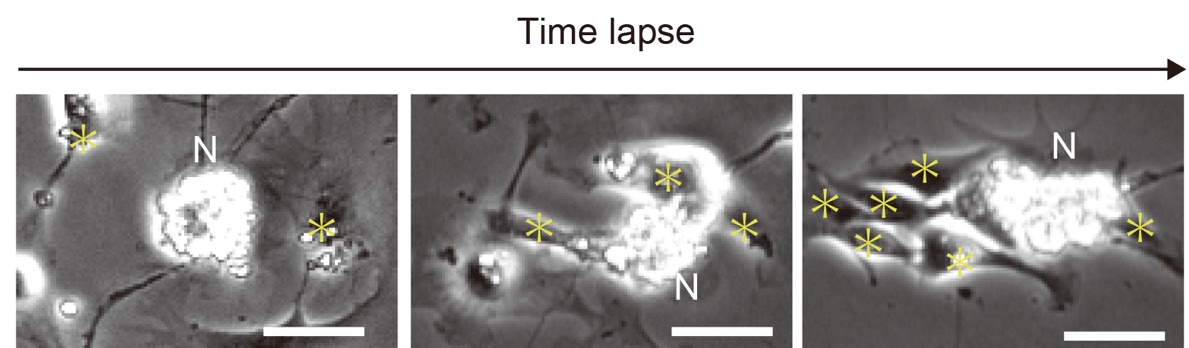
**

**Supplementary figure S5: IG27 cells attached to neuronal aggregates, growing around neuronal aggregate.**

Sequence of brightfield images from IG27 cells-primary neuron co-cultures via time lapse imaging. *, IG27 cell; N, neuronal aggregate. Scale bars, 10 µm. (See also Supplementary Movie S4).

**Supplementary Figure S6**

**
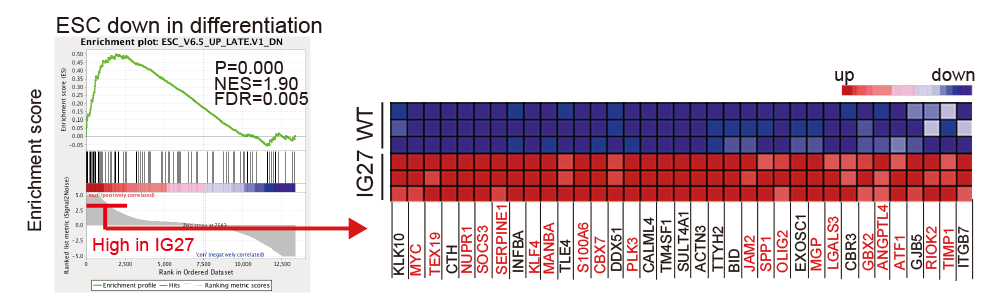

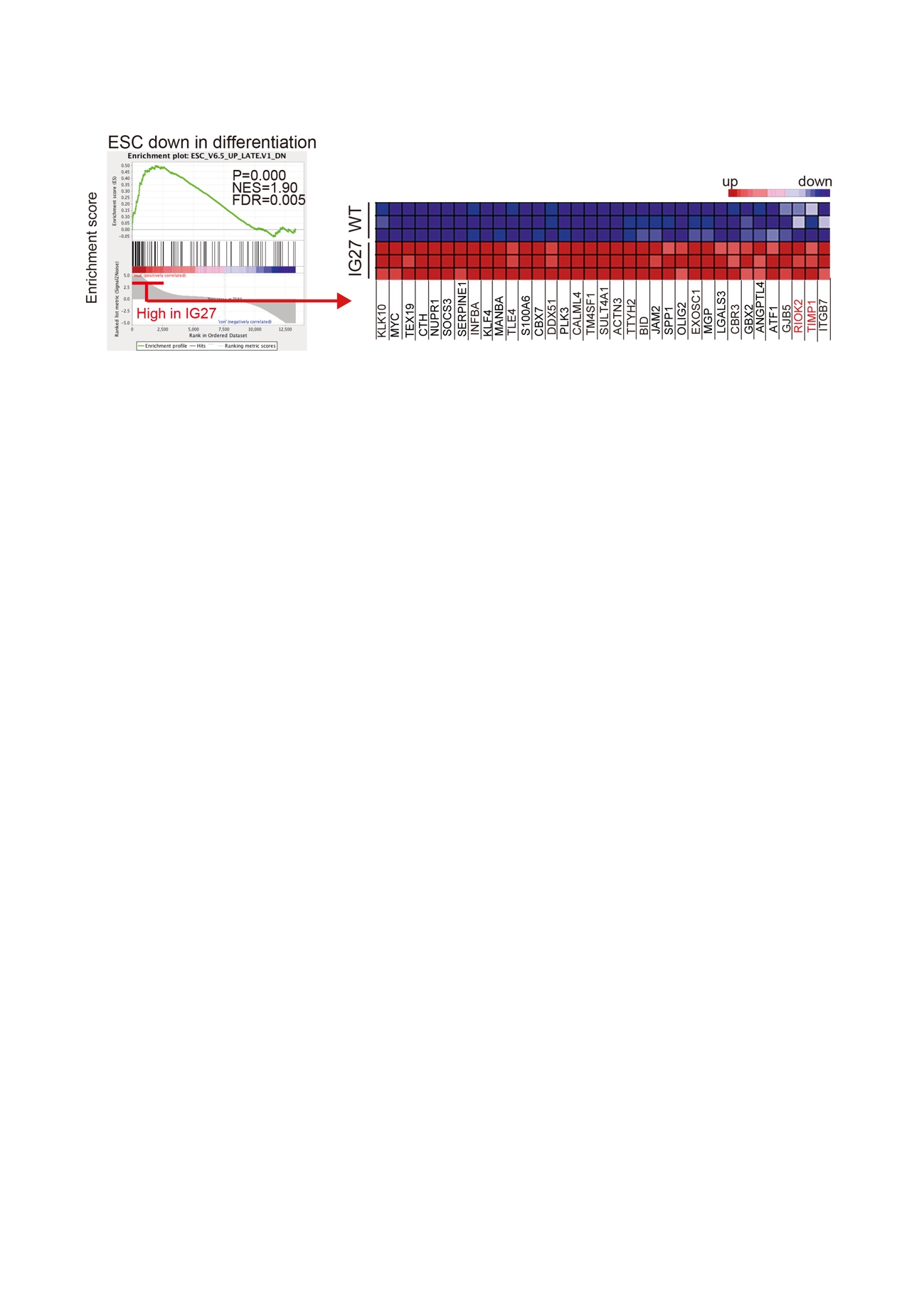
**

**Supplementary figure S6: Stemness gene signature in the IG27 cells versus WT OS3 glia cells by GSEA.**

Enrichment plot of genes downregulated during late stages of embryoid body differentiation from embryonic stem cells. Heat map indicates leading edge subset of upregulated genes (red); downregulated genes (blue). NES, normal enrichment score; FDR, false discovery rate. p-value was calculated with weighted Kolmogorov Smirnov test. Genes in red letters indicate elevated genes in human glioma cases in previous literatures.


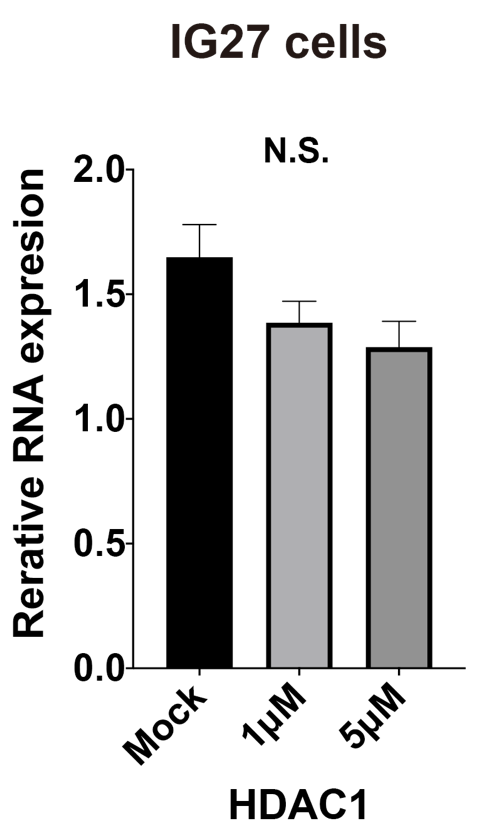
**Supplementary Figure S7**

**Supplementary figure S7: Relative Glut1 RNA expression in HDAC1-treated IG27 cells.**

Relative RNA expression levels of *Glut1* in each clone of histone deacetylase (HDAC) 1-treated IG27 cells by real-time RT-PCR. Data are presented as mean±SEM. n.s., not significant. *p* value determined by Mann-Whitney *U* test.

**Supplementary Figure S8**


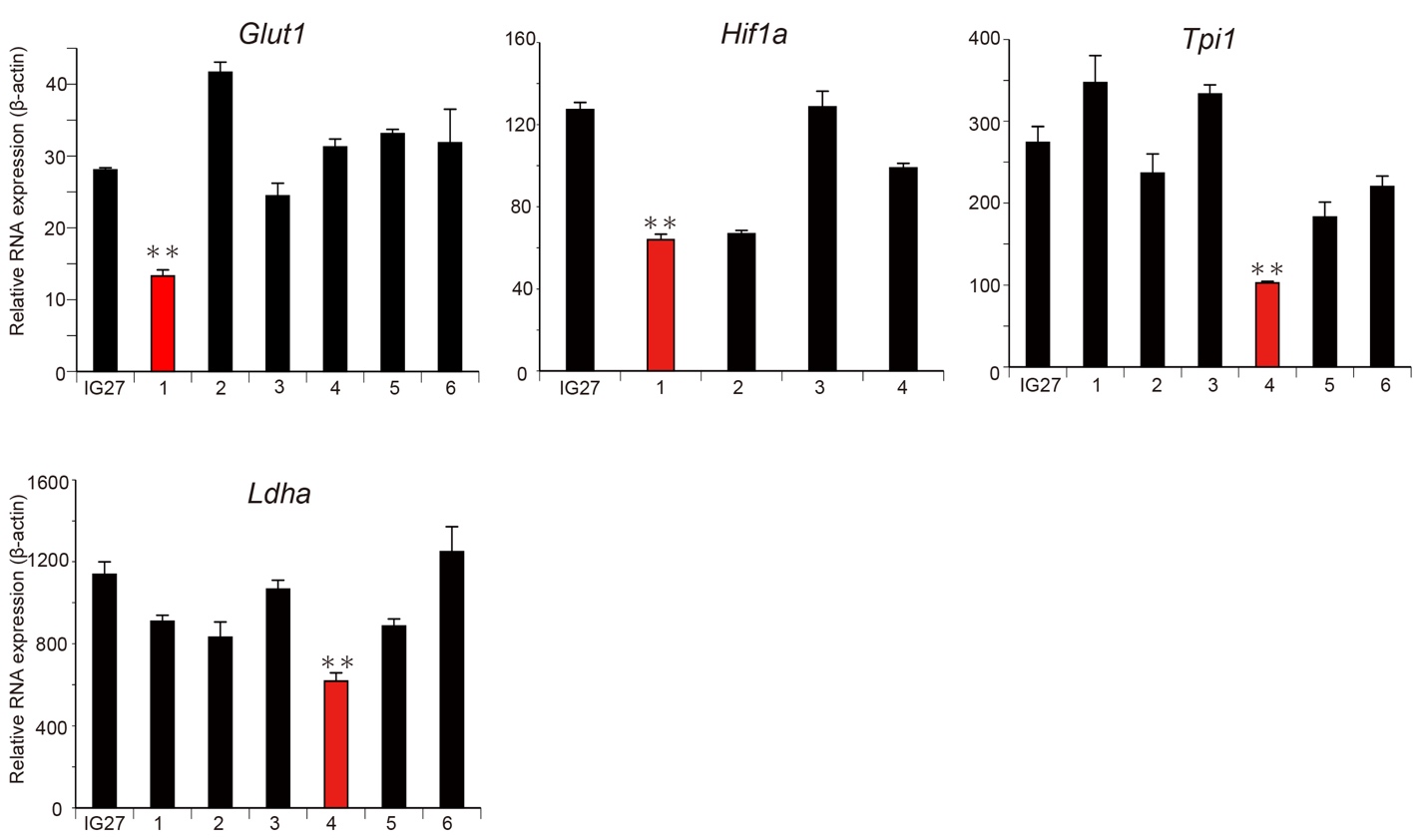


**Supplementary figure S8: Knockdown levels in each clone of stable IG27 cells carrying shRNA.**

Relative RNA expression levels of *Glut1, Hif1a, Tpi1, and Ldha* in each clone of IG27 cells carrying indicated shRNA compared with IG27 cells carrying shRNA negative by real-time RT-PCR. Number indicates clone number. The red colored clone was used *in vivo* injection. Data are presented as mean±SEM. **, p <0.01 compared with IG27 cells carrying shRNA negative. *p* value determined by Mann-Whitney *U* test.

**
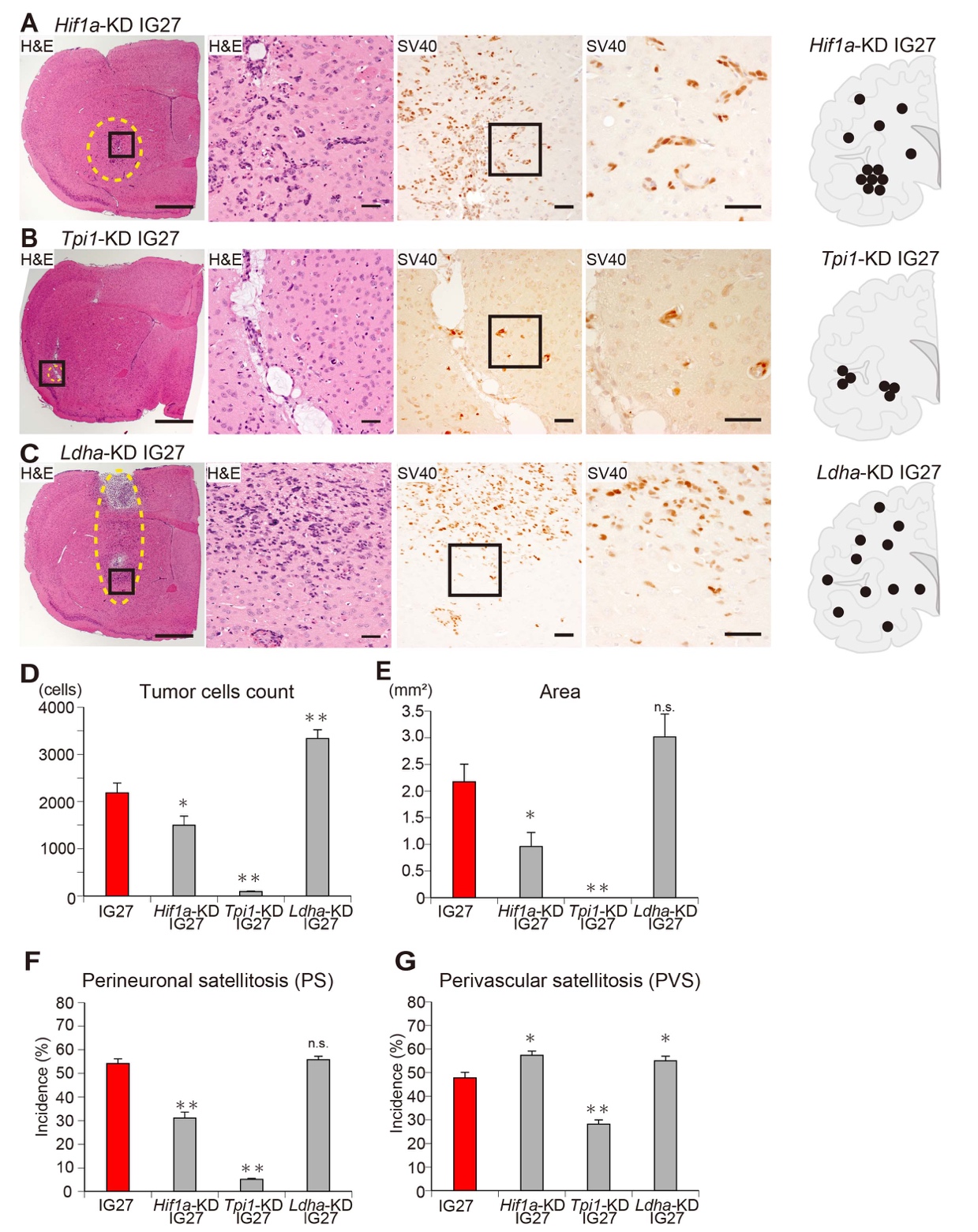
Supplementary Figure S9**

**Supplementary figure S9: Quantitative analysis for progression and invasion in gliomas of IG27 cells carrying *Hif1a-*, *Tpi1-*, and *Ldha-* shRNA.**

**(A-C)** H&E staining, IHC for SV40, and schematic distribution of glioma cells (black dots) at 4 weeks following transplantation of IG27 cells carrying shRNA-*Hif1a* (*Hif1a*-KD IG27) (***A***), shRNA-*Tpi1* (*Tpi1*-KD IG27) (***B***) and shRNA-*Ldha* (*Ldha*-KD IG27) (***C***) into mouse brains. Scale bars, 1 mm (*leftmost images*), 50 µm (*right images*).

(**D-G**) Quantitative analysis of tumor cell number (***D***), tumor expansion area (***E***), the incidence of PS (***F***) and PVS (***G***) at 4 weeks after transplantation of *Hif1a*-KD, *Tpi1*-KD, and *Ldha*-KD IG27 glioma cells. Tumor cell number (***D***) and tumor expansion area (***E***) were calculated in each cohort (n = 6, 7, and 14, respectively, five fields per mouse). The incidence of PS (***F***) and PVS (***G***) were counted in each cohort (n= 3 each, five fields per mouse). *Incidence*, number of neurons to which glioma cells attach out of the total number of neurons. Data are presented as mean±SEM. n.s., not significant. *, p<0.05 **, p <0.01 compared with IG27 cells carrying shRNA negative. *p* value determined by Mann-Whitney *U* test.

**Supplementary Figure S10**


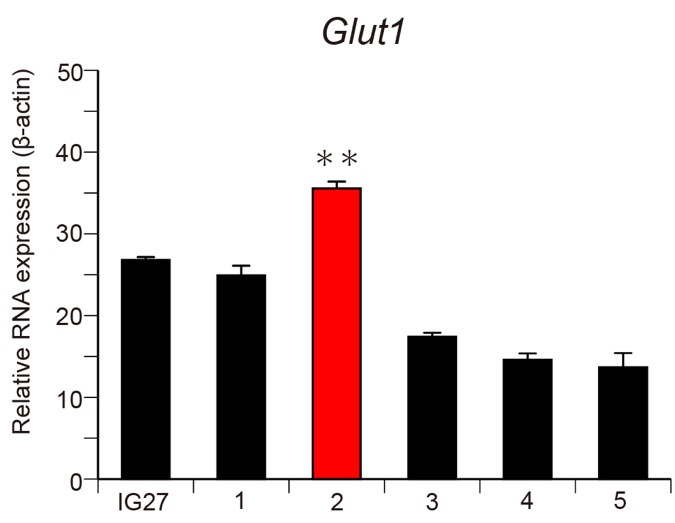


**Supplementary figure S10: Overexpression levels in clones of WT cells carrying *Glut1* cDNA expression vector.**

Relative RNA expression levels of *Glut1* in each clone of WT cells carrying *Glut1* cDNA vector compared with WT cells by real-time RT-PCR. Number indicates clone number. The red colored clone was used *in vivo* injection. Data are presented as mean ± SEM. **, p <0.01. p value determined by Mann-Whitney U test.

**Supplementary** **Figure S11**


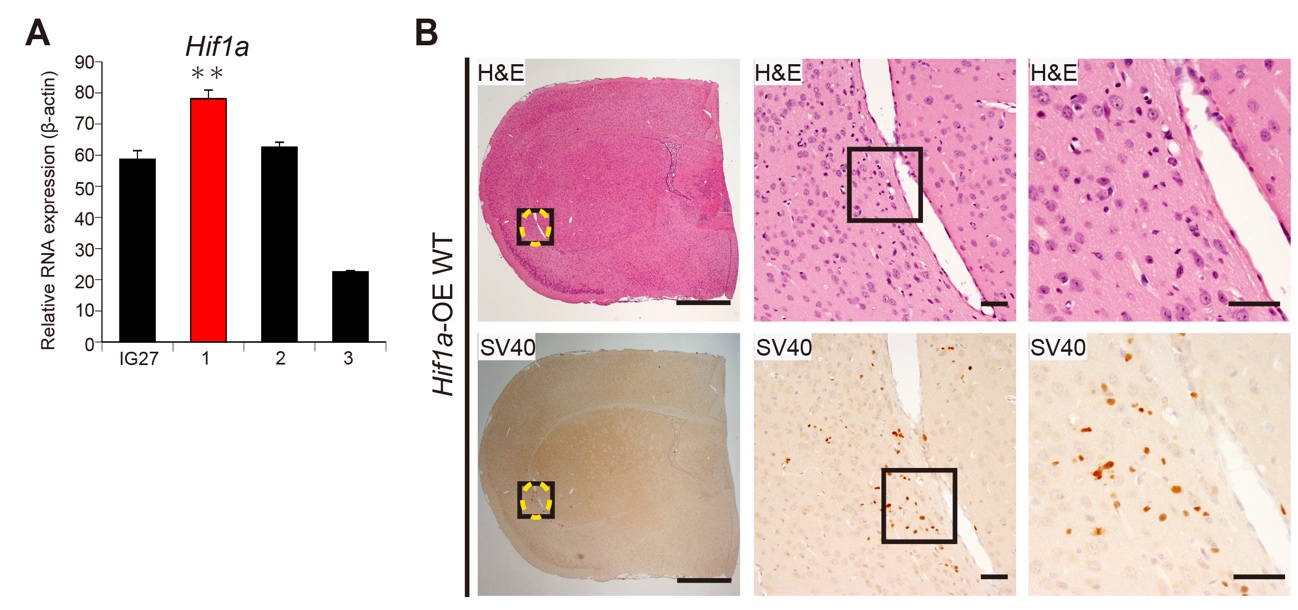


**Supplementary figure S11: *Hif1a* overexpression did not initiate diffuse invasion in WT cells.**

(**A**) Relative RNA expression levels of *Hif1a* in each clone of WT cells carrying *Hif1a* cDNA compared with WT cells by real-time RT-PCR. Number indicates clone number. The red colored clone was used *in vivo* injection. Data are presented as mean ± SEM. **, p <0.01. p value determined by Mann-Whitney U test.

(**B**) Representative images of Hif1a-OE WT glioma. H&E staining and IHC for SV40 at 4 weeks following transplantation of Hif1a-OE WT cells into mouse brains. The glioma infiltration area of Hif1a-OE WT glioma was much smaller than IG27- and Glut1-OE WT glioma　(See also Fig. 6A). Scale bars, 1 mm (*leftmost images*), 50 µm (*right images*).


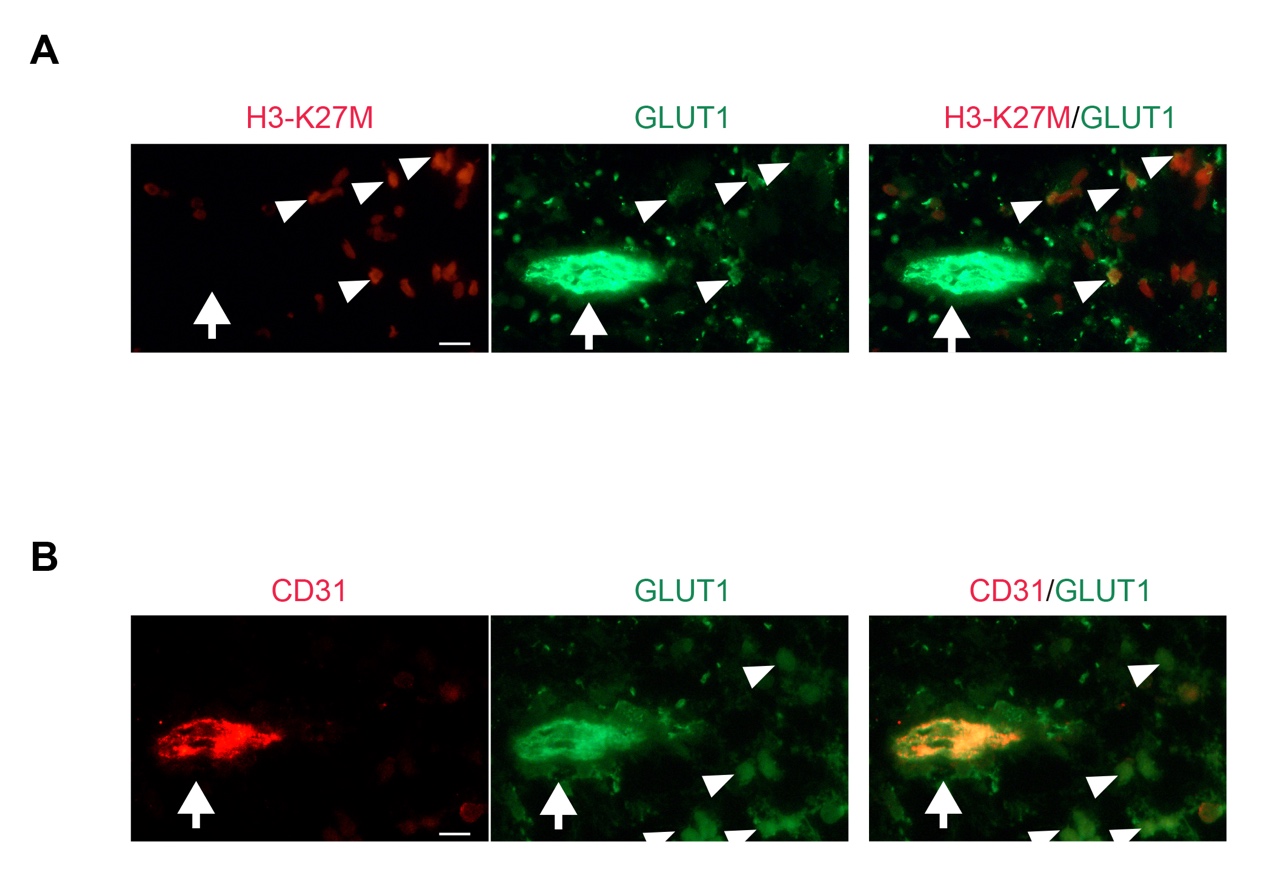
**Supplementary** **Figure S12**

**Supplementary figure S12: CD31, H3K27M, and GLUT1 expression in human H3K27M mutated glioma.**

(**A**) Double IF staining for histone H3 mutated K27M (red) and GLUT1 (green) in H3K27M-mutant diffuse glioma of human patient’s tissue. Scale bars, 10 μm.

(**B**) Double IF staining for CD31 (red) and GLUT1 (green) in H3K27M-mutant diffuse glioma of human patient’s tissue. Scale bars, 10 μm.

Arrows, vessel. Arrowheads, glioma cells.

**Supplementary** **Figure S13**


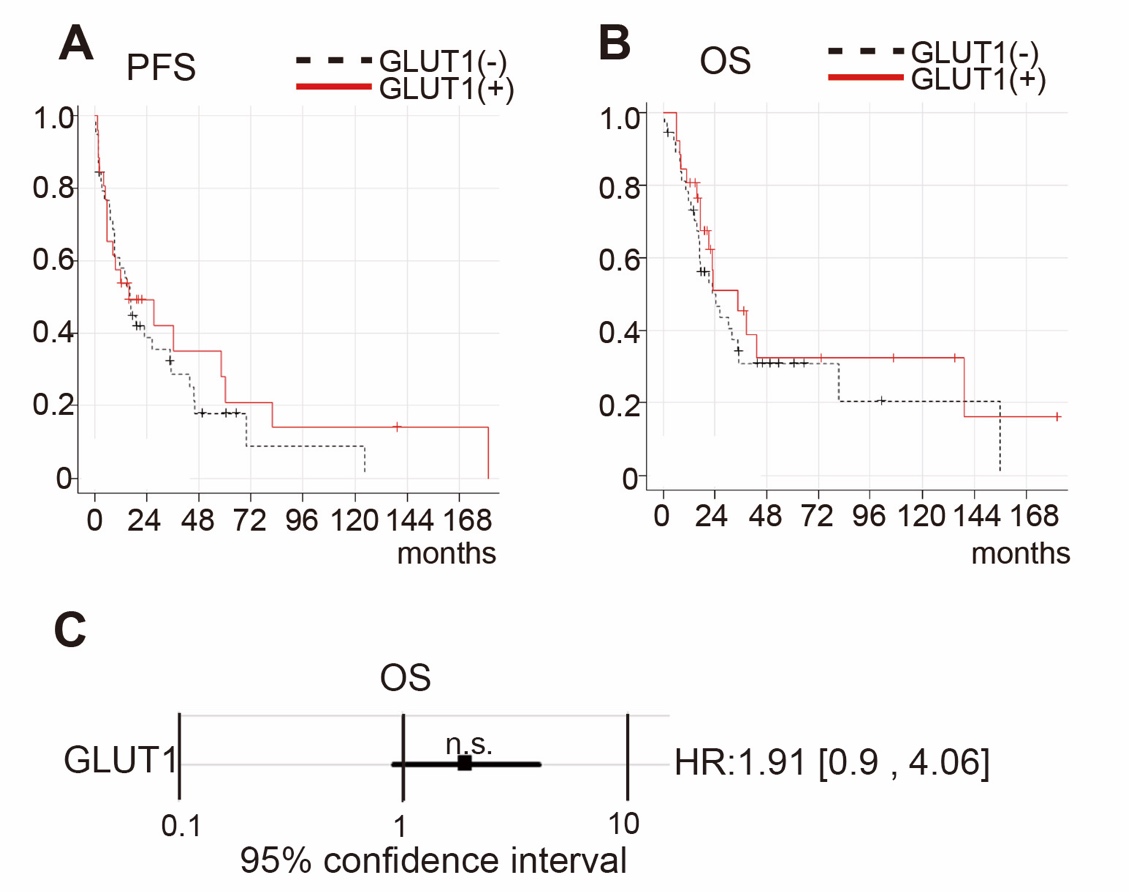


**Supplementary figure S13: GLUT1 expression is not associated with progression free survival (PFS) of univariate analysis and overall survival (OS) of univariate and multivariable analysis in human patients with gliomas.**

**(A and B)** Kaplan-Meier curves of PFS (**A**) and OS (**B**) in patients with glioma with the GLUT1 positive (red line) and negative expression (black dotted line).

**(C)** Multivariate analysis adjusted for age, recurrence, sex, and histological classification for OS. n.s., not significant.

**Supplementary Figure S14**


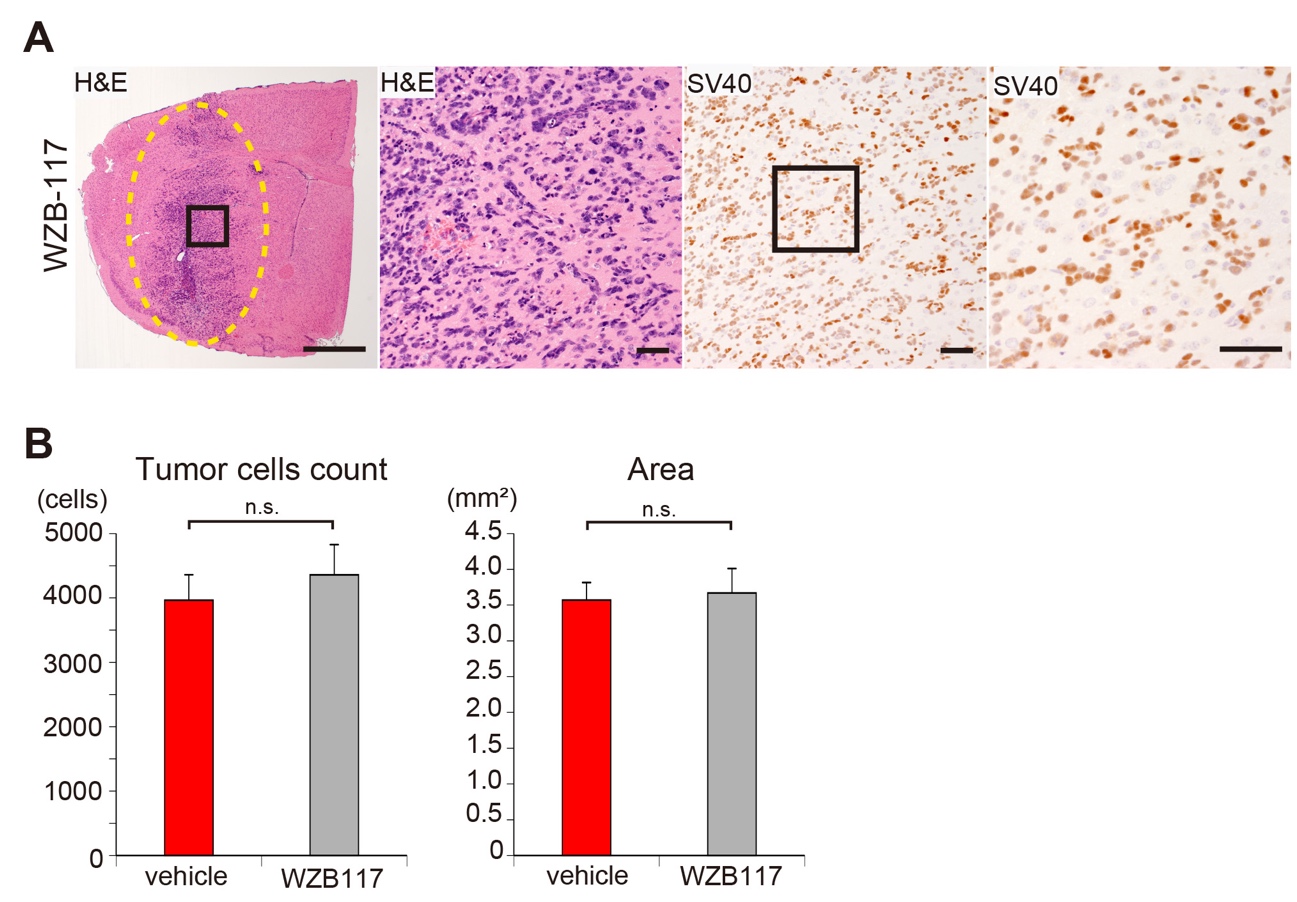


**Supplementary figure S14: Intraperitoneal injection of WZB117 resulted no reduction in the tumor cells and tumor expansion area.**

**(A)** Representative H&E staining and IHC for SV40 in IG27 gliomas of WZB117- treated mice. Scale bars, 1 mm (*leftmost image)*, 50 µm (*right images*).

(**B**) Quantitative analysis of tumor cell number and tumor expansion area in IG27 gliomas of WZB117- treated mice (n=10). *Incidence*, number of neurons to which glioma cells attach out of the total number of neurons. Bars represent mean ± SEM. n.s., not significant by Mann-Whitney test.

**Supplementary** **Figure S15**

**
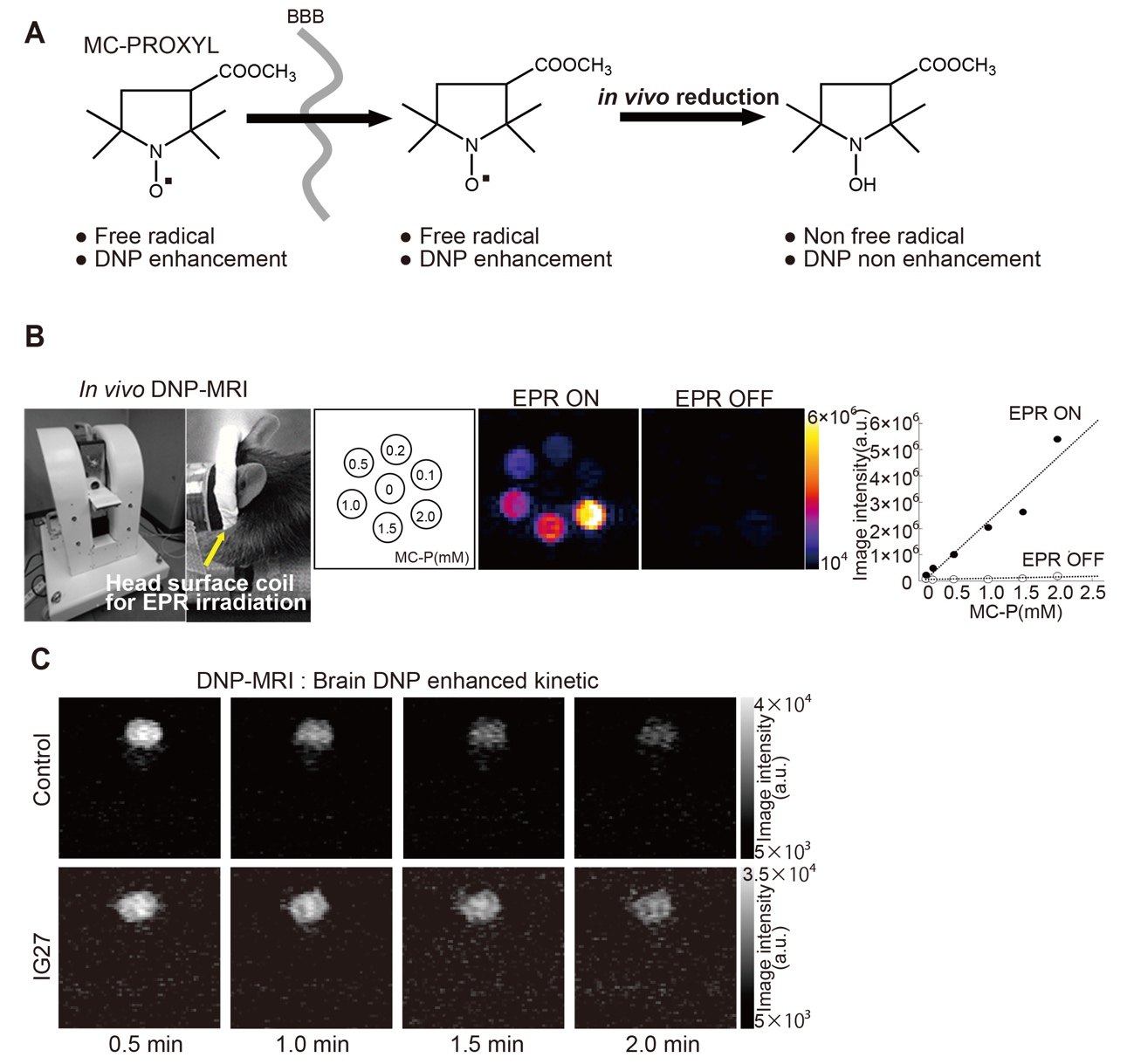
**

**Supplementary figure S15:** **DNP-MRI, but not MRI, is useful for detecting diffuse infiltration of IG27 cells.**

**(A)** Schematics of the molecular structure and one-electron reduction of MC-P in brain tissue after blood-brain barrier crossing.

**(B)** Pictures of *in vivo* DNP-MRI system and head surface coil for EPR irradiation. Schematics of MC-P phantom and DNP MR images with or without EPR irradiation.

**(C)** Time-course DNP-MR images of the head region in control (no-cell transplanted) and IG27-transplanted mice after injection of MC-P.

**Supplementary Methods**

**Glucose transporter Glut1 controls diffuse invasion phenotype with perineuronal satellitosis in diffuse glioma microenvironment**

Masafumi Miyai, Tomohiro Kanayama, Fuminori Hyodo, Takamasa Kinoshita, Takuma Ishihara, Hideshi Okada, Hiroki Suzuki, Shigeo Takashima, Zhiliang Wu, Yuichiro Hatano, Yusuke Egashira, Yukiko Enomoto, Noriyuki Nakayama, Akio Soeda, Hirohito Yano, Akihiro Hirata, Masayuki Niwa, Shigeyuki Sugie, Takashi Mori, Yoichi Maekawa, Toru Iwama, Masayuki Matsuo, Akira Hara, and Hiroyuki Tomita ^*^

**Supplementary Methods**

**Site-directed mutagenesis**

We purchased a plasmid containing the fully sequenced mouse H3f3a cDNA (Accession: BC106177, Clone ID: 6511787) (GE Healthcare Dharmacon Inc.). Primers containing the H3f3a-K27M site-directed mutation (AAG→ATG: K→M) sequence (Figure S1) were generated, amplified by PCR, and ligated into the EcoRI site using InFusion HD cloning kit (Clontech) according to the manufacturer's protocol.

**Cell proliferation**

Cell proliferation was assayed using the Cell Counting kit (Dojin Laboratories, Kumamoto, Japan). Cells were seeded on a 96-well plate with MEM-α supplemented with 10% FBS, 5 μg/mL bovine insulin, 0.2% D-glucose, and 0.1% penicillin-streptomycin solution and incubated in a humidified chamber at 37 °C and 5% CO2. Next, 10 µl of the thawed Cell Counting kit solution was added to each well, and the plates were incubated for 2 h at 37 °C and 5% CO2. The absorbance was measured using a 2104 EnVision Multilabel Reader (Perkin Elmer, USA) at 450 nm, with a reference wavelength of 600 nm.

**Western blotting**

Total protein was extracted from cultured cells using radioimmunoprecipitation assay (RIPA) buffer (Thermo 89900). Equal amounts of protein (75 μg) were separated on 10 % sodium dodecyl sulfate-polyacrylamide gel electrophoresis (SDS-PAGE) and blotted onto polyvinylidene difluoride (PVDF) membranes (Millipore). For detection of K27M mutant histone H3.3 and β-actin, we used #74829 and #4967 primary antibodies (Cell signaling), respectively, both at a 1:100 dilution. For secondary antibodies, we used horseradish peroxidase (HRP)–linked anti-rabbit or HRP–linked anti-goat (DAKO), both at a 1:2000 dilution. Detection was conducted using enhanced chemiluminescence.

**Histological analysis**

For IHC, sections were placed in citrate buffer (10 mmol/L, pH 6.0), autoclaved at 110 °C for 2 min 30 s for antigen retrieval, rinsed in PBS, and blocked in 3% hydrogen peroxide in methanol for 10 min to remove endogenous peroxidase. Non-specific binding sites were blocked in PBS containing 2% bovine serum albumin (Wako Pure Chemical, Osaka, Japan) for 40 min. Sections were incubated with primary antibodies overnight at 4 °C, washed for 5 min three times in PBS, and incubated with anti-rabbit and anti-mouse IgG antibodies labelled with peroxidase in Histofine Simple Stain MAX PO (Nichirei Corp., Tokyo, Japan) for 60 min at room temperature. Slides were washed three times (5 min for each wash) with PBS, developed with SIGMAFAST DAB for 2 min, counterstained with hematoxylin, dehydrated in a series of graded alcohols, and mounted in a Xylene-based mounting medium.

For IF, the primary antibody was used as for IHC. The slides were incubated with a secondary antibody conjugated with fluorescent proteins under a light-shielded condition at room temperature for 60 min. Then the slides were counterstained with DAPI solution (1:1000; WAKO, Japan) for 5 min, and covered with coverslips. All the antibodies are also listed in Table S5.

Tumor cells counted in the section which cut on injection point. Ki67-positive cells were counted in six high-power fields, and the average number of two or three mice was calculated.

**Scanning electron microscopy**

Mice were anesthetized and perfused with a solution comprising 2% glutaraldehyde, 2% sucrose, and 0.1 M sodium cacodylate buffer. Prior to perfusion, an incision was made in the right atrial appendage, and the neck was ligated with a silk suture. A perfusion pump was used for injection at a steady rate of 1 mL/min. The tissue was then harvested and diced. Three or four pieces of tissue (approximately 1 mm^3^ each) were immersed and fixed in the perfusion solution for 2 h, soaked overnight in a solution without glutaraldehyde, and then washed in an alkaline (0.03 M sodium hydroxide) sucrose (2%) solution. Specimens were then dehydrated through a graded ethanol series and subjected to the frozen fracture method for scanning electron microscopy (S-4800; Hitachi, Tokyo, Japan), as described previously^1^.

**HDAC treatment**

HDAC1 (SignalChem, H83-39G) were administrated 1μM, 5μM with mock into the IG27 cells for 48 hours.

**Lactate assay**

Supernatant samples were obtained from cells that were cultured for 24 h and mixed with solutions included in the Lactate assay kit-WST (Dojindo Molecular Technologies, Inc., Kumamoto, Japan) according to the manufacturer's protocol. MEMα medium without phenol red was used in this experiment. The mixture was incubated at 37 ^o^C for 30 min. Finally, the absorbance was set to 450 nm and measured using a microplate reader. Lactate concentration was determined using a calibration curve.

**Microarray analysis**

Total RNA was isolated from cells using the Maxwell RSC simplyRNA Tissue Kit (Promega Corp., Fitchburg, Wisconsin, USA; cat. #AS1340). Cy3-labelled probes were prepared from total RNA using the Low Input Quick Amp Labeling Kit 1-color (Agilent Technologies, Santa Clara, California, USA; #5190-2305) and hybridized with a microarray slide (SurePrint G3 Mouse GE 8 × 60 K Microarray; Agilent Technologies) for 17 h at 65 ℃. Next, the slide was washed and scanned with a microarray scanner (ArrayScan, Agilent Technologies) to obtain the probes’ fluorescent signal and processed for digitization using Feature Extraction software (Agilent), and analyzed with GeneSpring GX software (Agilent) for gene expression analysis. Moderated t-tests were used to reveal significant differences in gene expression between IG27 and WT cells. In all analyses, P ≤ 0.05 was considered statistically significant. Further, GSEA was used to analyze pathway enrichment.

**Metabolome analysis**

Culture medium was removed from the dish and cells were washed twice in 5% mannitol solution (10 mL first and then 2 mL). Cells were then treated with 800 μL of methanol and 550 μL of Milli-Q water containing internal standards (H3304-1002, HMT, Inc., Tsuruoka, Japan). The metabolite extract was transferred into a microfuge tube and centrifuged at 2,300 ×g and 4ºC for 5 min. Next, the upper aqueous layer was centrifugally filtered through a Millipore 5-kDa cutoff filter at 9,100 ×g and 4ºC for 120 min to remove proteins. The filtrate was centrifugally concentrated and re-suspended in 50 μL of Milli-Q water for CE-MS analysis. Anionic compounds were analyzed in the positive and negative modes of CE-MS/MS. To obtain peak information including m/z, migration time (MT), and peak area, detected peaks by CE-MS/MS were extracted using automatic integration software (MasterHands, Keio University, Tsuruoka, Japan and MassHunter Quantitative Analysis B.04.00, Agilent Technologies, Santa Clara, CA, USA, respectively). The peaks were annotated with putative metabolites from the HMT metabolite database based on their MTs in CE and m/z values determined by MS/MS. The tolerance range for the peak annotation was configured at ± 0.5 min for MT and ±10 ppm for m/z. In addition, concentrations of metabolites were calculated by normalizing the peak area of each metabolite with respect to the area of the internal standard and by using standard curves, which were obtained by three-point calibrations.

**Glucose Uptake assay**

Cells were treated as indicated for 24 h, then seeded in a 96-well plate (5.0 × 10^3^ cells/well) and cultured for overnight. The culture medium was removed and cells were washed with PBS. Then, cells were incubated with fresh-prepared 500 mM2-deoxyglucose (2-DG, 50 μl/well) for 20 min at room temperature. The uptake process was stopped and neutralized, and luciferase activities were measured by Glucose Uptake-Glo Assay (Promega). Rate of glucose uptake was analyzed according to the manufacturer’s instructions.

**Seahorse XF glycolysis stress test**

Glycolytic rates were measured using the XF Extracellular Flux Analyzer (Seahorse Bioscience, North Billerica, MA). WT and IG27 cells were seeded at a density of 2.0 × 10^4^ cells/well using a Seahorse plate coated with 0.2% gelatin and cultured for overnight. Cells were equilibrated with XF Base media (Seahorse, catalog number 102353-100) at 37 °C for 1 h in an incubator lacking CO2. Glycolysis was measured using Seahorse XF Glycolysis Stress Test Kit (Seahorse, catalog number 103020-100) according to the manufacturer’s protocol followed by sequential treatments with glucose (10 mM), oligomycin (1.0 μM), and 2-DG (50 mM).

**Chromatin isolation and Chip-qPCR**

Chromatin isolation and ChIP of cultured cells was performed according to the manufacturer’s protocol with minor modifications using the SimpleChIP Plus Enzymatic Chromatin IP Kit (Cell Signaling Technology). Briefly, WT and IG27 cells were fixed with 1% formaldehyde for 10 minutes and the cross-linking reaction stopped by addition of glycine to a final concentration of 125 mM. Cells were then washed, lysed, and then sonicated. The supernatant was pre-cleared with Protein-G beads and incubated overnight at 4 °C with histone H3 (acetyl K27)-specific rabbit monoclonal antibody (abcam), or rabbit IgG monoclonal antibody (abcam) as negative control. The DNA–protein complexes were then washed and eluted. Cross-linking was reversed, and recovered DNA was subjected to quantitative PCR using primers of the *Glut1* gene promoter: #1:forward 5’- TACACCCCAGAACCAATGGC -3’ and reverse 5’- CCCGTAGCTCAGATCGTCAC -3’, #2: forward 5’- CAAGAGCAGAGGCTTGCTTG -3’ and reverse 5’- AGAACGGACGCGCTGTAACT -3’.

**Phantom experiments**

Seven tubes of phantom, each tube (4-mm i.d.) containing 0-2 mM MC-P in PBS were used (Supplementary Figure 11). The scanning conditions for *in vivo* DNP-MRI were as follows: EPR irradiation power, 7W; flip angle (FA), 90°; repetition time (T_R_) × echo time (T_E_) × EPR irradiation time (TEPR), 500 × 25 × 250 ms; accumulation number, 2; slice thickness, 20 mm, including the whole thickness of the mouse; phase-encoding steps, 32; field of view (FOV), 40 × 40 mm; and matrix size, 64 × 64 after reconstruction. The DNP-MRI data were analyzed using Image J software.

**DNP-MRI imaging**

DNP-MRI images of the phantom with or without electron paramagnetic resonance (EPR) irradiation to induce the DNP effect in various concentrations of MC-P are shown (Supplementary Figure 11). DNP-MR signal intensity with EPR irradiation linearly increased with concentrations of MC-P up to 2 mM (Supplementary Figure 11). In contrast, image intensity of the phantom without EPR irradiation was very low due to the low magnetic field of the DNP-MRI system (15 mT) (Supplementary Figure 11).

When we explain it in detail, *In vivo* redox molecular imaging was performed using a low magnetic field, *in vivo* DNP-MRI system (Keller-Japan redox Inc.) (Supplementary Figure 11). The external magnetic field (B_0_) for EPR irradiation and MRI was fixed at 15 mT, and the respective radiofrequencies were 455 MHz and 683 kHz. A one-turn circle surface coil for brain imaging was constructed for EPR irradiation during head imaging (Supplementary Figure 11). Mice were anesthetized with 1.5% isoflurane and then secured in a head position on a special holder with adhesive skin tape. During the procedure, the mouse body temperature was kept at 37 ± 1 °C with a heating pad. DNP-MRI scanning of the head started immediately after intravenous injection of MC-P (150 mM MC-P in half normal saline (0.45% saline), 7.5 μL/g body weight). Pharmacokinetic images were obtained at 0.5 ,1, 1.5, 2, 2.5, 3, 3.5, 4, 4.5, and 5 min after administration. Normal MR images with 1 and 10 accumulations were obtained without EPR irradiation. The *in vivo* redox metabolic map was obtained by the slope of the enhanced DNP image intensity of each pixel from four pharmacokinetic images using a custom Excel macro program. The scanning conditions were as follows: power of EPR irradiation, 7W; FA, 90°; T_R_ × T_E_ × TEPR, 500 × 25 × 250 ms; accumulation number, 2; slice thickness, 20 mm, including the whole thickness of the mouse; phase-encoding steps, 32; FOV, 40 × 40 mm; and matrix size, 64 × 64 after reconstruction. Data were analyzed using Image J software.

**1.5-T MRI**

MRI measurements were performed at a 1.5-T scanner controlled by Medalist software (JAPAN Redox Inc., Fukuoka, Japan). A series of T_1_- and T_2_-weighted fast spin echo (T_1_: TR, 500 ms; TE, 12 ms; FA, 90˚; N_EX_, 2; and T_2_: TR, 3000 ms; TE, 90 ms; FA, 90˚; N_EX_, 2; echo factor, 4) were employed. Other imaging parameters were as follows: image resolution, 256 × 128 zero-filled to 256 × 256 (0.125 mm resolution); FOV, 4 × 4 cm; slice thickness, 2.0 mm; 0.5-mm gap; and number of slices, 5.

**Statistical Analysis for human data**

For human glioma experiments, data were analyzed using SPSS version 22 (IBM Japan Ltd., Tokyo, Japan) and R software version 3.5.1 (www.r-project.org). The characteristics of patients were summarized by median values with 25th and 75th percentiles for continuous variables (Supplementary Table 4). Frequencies and percentages are shown for categorical variables. Cumulative probability of PFS and OS were generated using Kaplan-Meier estimates, which were used to assess differences between positive and negative groups of GLUT1 protein expression in human glioma tissues. Subjects in Kaplan-Meier estimates were censored at the earlier of time of last contact or at 168 months follow-up of patients. Multivariable Cox proportional hazard models were used to assess the relationship between outcome (PFS and OS) and GLUT1 protein expression. Adjustments were made for age, recurrence, sex and histological classification, and hazard ratios and 95 percent confidence intervals (95% CI) were calculated using Cox proportional hazard models. We limited the covariates in the model to 4 confounders and one gene to avoid overfitting. The selection of covariates was made a priori according to expert opinion and previous literature. All analyses used a 5% two-sided significance level, nonsignificant was indicated “n.s.”, and significance was indicated as *P < 0.05 and **P < 0.01.

**Reference**

1) Ando, Y., Okada, H., Takemura, G., Suzuki, K., Takada, C., Tomita, H., Zaikokuji, R., Hotta, Y., Miyazaki, N., Yano, H., et al. (2018). Brain-Specific Ultrastructure of Capillary Endothelial Glycocalyx and Its Possible Contribution for Blood Brain Barrier. Sci Rep 8, 17523.

**Supplementary Movie legends:**

Movie 1: Time lapse movie showing co-culture of WT cells and primary neurons.

Movie 2: Time lapse movie showing co-culture of IG27 cells and primary neurons.

Movie 3: Time lapse movie showing the cell tracking of WT and IG27 cells co-cultured with primary neurons.

Movie 4: Time lapse movie showing the accumulation, division, and growth of IG27 cells around a neuronal aggregate.

**Supplementary Table S1: All the materials used in this study**

| Materials | Source | identifier |
| --- | --- | --- |
| Antibodies | | |
| Rabbit monoclonal anti-Histone H3 (mutated K27 M) antibody [EPR18340] (1:200) | Abcam, Cambridge, UK | Cat# ab190631 |
| Mouse monoclonal anti-Histone H3 (tri methyl K27) antibody [mAbcam 6002] (1:200) | Abcam, Cambridge, UK | Cat# ab6002 |
| Rabbit monoclonal anti-Histone H3 (acetyl K27) antibody [EP865Y] (1:200) | Abcam, Cambridge, UK | Cat# ab45173 |
| Rabbit monoclonal anti-Histone H3 (acetyl K27) antibody [EP16602]-ChIP Grade (1.0 µg) | Abcam, Cambridge, UK | Ca# ab177178 |
| Rabbit monoclonal anti-Ki67 [SP6] antibody (1:100) | Abcam, Cambridge, UK | Cat# ab16667 |
| Mouse monoclonal anti-SV40 T-antigen antibody [PAb416]  (1:200) | Abcam, Cambridge, UK | Cat# ab16879 |
| Mouse monoclonal anti-NeuN clone A60 antibody (1:1000) | Millipore,Temecula, California,USA | Cat# MAB377 |
| Rabbit monoclonal CD31 (PECAM-1) (D8V9E) XP® antibody (1:100) | Cell Signaling Technology, Danvers, MA, USA | Cat# 77699 |
| Anti-Mouse CD32(PECAM-1)/Endothelial Cell Marker Rat Monoclonal Antibody (1:50) | Dianova GmbH, Hamburg,Germany | Ca# DIA 310 |
| Chicken polyclonal anti-MAP2 antibody (1:500) | Abcam, Cambridge, UK | Cat# ab5392 |
| Rabbit monoclonal anti-Glucose Transporter GLUT1 antibody [EPR3915] (1:2000) | Abcam, Cambridge, UK | Cat# ab115730 |
| Mouse monoclonal anti-Glut1(A-4) antibody (1:100) | Santa Cruz Biotechnology, Santa Cruz, CA, USA | Ca# sc-377228 |
| Mouse monoclonal anti-p53(Pab 246) antibody (1:100) | Santa Cruz Biotechnology, Santa Cruz, CA, USA | Cat# sc-100 |
| CD140a (PDGFRA) Monoclonal antibody (APA5) (1:250) | eBioscience, San Diego, CA, USA | Ca# 14-1401-81 |
| Rabbit IgG monoclonal [EPR25A] Isotype Control (1.667µg) | Abcam, Cambridge, UK | Ca# ab172730 |
| Goat Anti-Rabbit IgG H&L (Alexa Fluor® 405) preadsorbed (1:200) | Abcam, Cambridge, UK | Cat# ab175654 |
| Goat Anti-Mouse IgG H&L (Alexa Fluor® 488) preadsorbed(1:200) | Abcam, Cambridge, UK | Cat# ab150117 |
| Goat Anti-Rabbit IgG H&L (Alexa Fluor® 488) preadsorbed (1:200) | Abcam, Cambridge, UK | Cat# ab150081 |
| Goat Anti-Chiken IgY H&L (Alexa Fluor® 488) preadsorbed(1:200) | Abcam, Cambridge, UK | Cat# ab150173 |
| Goat Anti-Rabbit IgG H&L (Alexa Fluor® 594) preadsorbed(1:200) | Abcam, Cambridge, UK | Cat# ab150084 |
| Goat Anti-Mouse IgG H&L (Alexa Fluor® 594) preadsorbed(1:200) | Abcam, Cambridge, UK | Cat# ab150120 |
| Goat Anti-Chiken IgY H&L (Alexa Fluor® 594) preadsorbed(1:200) | Abcam, Cambridge, UK | Cat# ab150176 |
| Rabbit Polyclonal RFP Antibody Pre-adsorbed (1:200) | Rockland Immunochemicals, Gilbertsville, PA, USA | Cat# 600-401-379 |
| Rabbit IgG (Control Antibody) | Vector Laboratories, Burlingame,CA,USA | Cat# I-1000 |
| Mouse IgG (Control Antibody) | Vector Laboratories, Burlingame,CA,USA | Cat# I-2000 |
| Histofine Simple Stain Mouse MAX-PO (R) | Nichirei Corp, Tokyo, Japan | Cat# 414341 |
| Histofine Mouse Stain kit | Nichirei Corp, Tokyo, Japan | Cat# 414322 |
| Histofine Simple Stain MAX PO (R) | Nichirei Corp, Tokyo, Japan | Cat# 424142 |
| Histofine Simple Stain MAX PO (M) | Nichirei Corp, Tokyo, Japan | Cat# 424132 |
| Bacterial and Virus Strains |  |  |
| HIF-1α shRNA (m) Lentiviral Particles | Santa Cruz Biotechnology, Santa Cruz, CA, USA | Cat# sc-35562-V |
| Glut1 shRNA (m) Lentiviral Particles | Santa Cruz Biotechnology, Santa Cruz, CA, USA | Cat# sc-35494-V |
| TIM shRNA (m) Lentiviral Particles | Santa Cruz Biotechnology, Santa Cruz, CA, USA | Cat# sc-37173-V |
| LDH-A shRNA (m) Lentiviral Particles | Santa Cruz Biotechnology, Santa Cruz, CA, USA | Cat# sc-45898-V |
| Biological Samples |  |  |
| Mouse brain tumor via transplant mouse glioma cell | This paper | N/A |
| Human brain tumor samples | This paper | N/A |
| Chemicals, Peptides, and Recombinant Proteins |  |  |
| Glucose Transporter Inhibitor IV, WZB117 | EMD Millipore, Billerica, MA,USA | Ca# 400036-25MGCN |
| BAY-876 | Sigma-Aldrich Chemical Company, St. Louis,  MO, USA | Ca# SML1774-25MG |
| Polyinosinic-Polycytidylic acid | Sigma-Aldrich Chemical Company, St. Louis,  MO, USA | Cat# P1530 |
| X-Gal（5-Bromo-4-Chloro-3-Indolyl-β-D-Galactoside） | Takara, Tokyo, Japan | Cat# 9031 |
| PrimeSTAR® Max DNA Polymerase | Takara, Tokyo, Japan | Cat# R045A |
| KOD FX Neo | Toyobo Life Science, Tokyo, Japan | Cat# KFX-201 |
| - Cellstain - DAPI solution | Dojindo Molecular Technologies, Kumamoto, Japan | Cat# D523 |
| SIGMAFAST™ 3,3′-Diaminobenzidine tablets tablet, To prepare 5 mL | Sigma-Aldrich Chemical Company, St. Louis,  MO, USA | Cat# D4293 |
| Lipofectamine LTX and PlusTM Reagent | Invivogen, Carlsbad, CA, USA | Cat# 15338-100 |
| Geneticin(G418) | Life Technologies, Carlsbad, CA, USA | Cat# 10131-035 |
| Puromycin | Invivogen, San Diego, CA, USA | Cat# ant-pr-1 |
| Hygromycin B Gold | Invivogen, San Diego, CA, USA | Cat# ant-hg-1 |
| Zeocin | Invivogen, San Diego, CA, USA | Cat# ant-zn-1 |
| Hematoxylin | Mayer P: Zschr.wiss.Mikrosk. 20:409.1904. | N/A |
| Eosin Y | FUJIFILM Wako Pure Chemical Co. (Osaka, Japan) | Cat# 058-00062 |
| MEM α GlutaMAX™ | Thermo Fisher Scientific Waltham, MA, USA | Cat# 32561-037 |
| MEM Alpha | Thermo Fisher Scientific Waltham, MA, USA | Ca# 41061-029 |
| Seahorse XF Base Medium - phenol free, | Agilent Technologies, Santa Clara, CA, USA | Ca# 103335-100 |
| D-(+)-Glucose solution | Sigma Chemicals, Poole, Dorset, UK | Cat# G8644-100ML |
| Insulin solution from bovine pancreas | Sigma Chemicals, Poole, Dorset, UK | Cat# I0516-5ML |
| Fetal Bovine Serum (FBS) | BioWest, Miami, FL, USA | Cat# S1530 |
| GlutaMAX™ I | Life Technologies, Carlsbad, CA, USA | Cat# 35050-061 |
| Dulbecco's Modified Eagle Medium (DMEM) (1×) | Thermo Fisher Scientific, Waltham, MA, USA | Cat# 11965-092 |
| N-2 Supplement, 100× | Thermo Fisher Scientific, Waltham, MA, USA | Cat# 17502-048 |
| Recombinant Human Epidermal Growth Factor (Hu EGF) | Thermo Fisher Scientific, Waltham, MA, USA | Cat# PHG0311 |
| Geltrex™ LDEV-Free Reduced Growth Factor Basement Membrane Matrix | Thermo Fisher Scientific, Waltham, MA, USA | Cat# A1413202 |
| Dulbecco's Phosphate Buffered Saline | Sigma Chemicals, Poole, Dorset, UK | Cat# D8537-500ML |
| Penicillin-Streptomycin Solution | Sigma Chemicals, Poole, Dorset, UK | Cat# P4458 |
| 0.25% Trypsin-EDTA (1×) | Thermo Fisher Scientific, Waltham, MA, USA | Cat# 25200-056 |
| Neuron Dissociation Solutions Kit | FUJIFILM Wako Pure Chemical Co. ,Osaka, Japan | Cat# 291–78001 |
| Neurobasal medium | Thermo Fisher Scientific, Waltham, MA, USA | Cat# 21103049 |
| Neuron Culture Medium | FUJIFILM Wako Pure Chemical Co. ,Osaka, Japan | Cat# 148-09671 |
| B-27 Supplement (50X), serum free | Thermo Fisher Scientific, Waltham, MA, USA | Cat# 17504044 |
| Ara-C | Sigma Chemicals, Poole, Dorset, UK | Cat# C1768 |
| PowerUp SYBR Green Master Mix | Thermo Fisher Scientific, Waltham, MA, USA | Cat# A25742 |
| Glutaraldehyde | Nakarai chemicals Ltd, Kyoto, Japan | Cat# 17025 |
| Sucrose | FUJIFILM Wako Pure Chemical Co. ,Osaka, Japan | Cat# 196-00015 |
| Cacodylic acid sodium salt | Nakarai chemicals Ltd, Kyoto, Japan | Cat# 06516 |
| Sodium hydroxide | FUJIFILM Wako Pure Chemical Co. ,Osaka, Japan | Cat# 198-13765 |
| Critical Commercial Assays |  |  |
| SuperScript™ III First-Strand Synthesis System | Thermo Fisher Scientific, Waltham, MA, USA | Cat# 18080051 |
| RNeasy Mini Kit | Qiagen, Valencia, CA, USA | Cat# 74104 |
| Lactate Assay Kit - WST | Dojindo Molecular Technologies, Kumamoto, Japan | Cat# L256 |
| Glucose Uptake-Glo Assay | Promega, Southampton, UK | Ca# J1341 |
| SimpleChIP® Plus Sonication ChIP Kit 4℃ and RT Reagents | Cell Signaling Technology, Danvers, MA, USA | Ca# 57976 |
| Cell Counting Kit-8 | Dojindo Molecular Technologies, Kumamoto, Japan | Cat# 347-07621 |
| Agilent Seahorse XFp Glycolysis Stress Test Kit | Agilent Technologies, Santa Clara, CA, USA | Ca# 103017-100 |
| Deposited Data |  |  |
| RNA microarray raw data | This paper | GEO: GSE126725 |
| Experimental Models: Cell Lines |  |  |
| OS3 | Asakura K, Neurosci Lett 1998 258:21-4 | Cat# RCB1593 |
| Experimental Models: Organisms/Strains |  |  |
| CAnN.Cg-Foxn1nu/CrlCrlj | Charles River Laboratories, Japan |  |
| C3H/HeN | Charles River Laboratories, Japan |  |
| Oligonucleotides |  |  |
| Fragment 1 infusion primers overhanging with H3f3a-K27M mutation  F_1:CAGTGTGGTGGAATTCATGGCTCGTACAAAGCAGA  R_1:AAGCAACTGGCTACAAAAGCCGCTCGCATGAGTGCG | This paper | N/A |
| Fragment 2 infusion primers overhanging with H3f3a-K27M mutation  F_2 :GCTCGCATGAGTGCGCCCTCTACTGGAGGGGTGAA  R_2:GATATCTGCAGAATTCTTGATTACAAGGATGACGACGATAAGAAGCACGTTCTCCACGT | This paper | N/A |
| Primers for real-time RT-PCR, see Table S5. | This paper | N/A |
| Recombinant DNA |  |  |
| fully sequenced Mouse H3f3a cDNA (Accession: BC106177 Clone ID: 6511787) | GE Healthcare Dharmacon Inc. Lafayette, CO, USA | N/A |
| pIRES vector | Takara, Tokyo, Japan | Cat# No 631605 |
| Software and Algorithms |  |  |
| GSEA | subramanian, 2005 | http://software.broadinstitute.org/gsea/index.jsp |
| Prism 6.0d | GraphPad Software Inc., San Diego CA | NA |
| PrimerBLAST | NCBI | https://www.ncbi.nlm.nih.gov/tools/primer-blast/ |
| DAVID | NCBI | https://david.ncifcrf.gov |
| CellSens software | Olympus, Tokyo, Japan |  |
| Cell Activision | Yokogawa Electric Corporation, Tokyo, Japan |  |
| The Agilent Seahorse XFp | Agilent Technologies, Santa Clara, CA, USA | Ca# S7802A |
| IBMSPSS version 22 | IBM Japan Ltd, Tokyo, Japan |  |
| R software version 3.5.1 | The R Foundation. | www.r-project.org |
| Image J software | Schneider et al., 2012 |  |
| Medalist software | JAPAN Redox Inc. , Fukuoka, Japan |  |
| Other |  |  |
| 96 Well Cell Culture Cluster | Costar, Cambridge, MA, USA | Cat# 3596 |
| 24 Well Cell Culture Cluster | Costar, Cambridge, MA, USA | Cat# 3526 |
| 12 Well Cell Culture Cluster | Costar, Cambridge, MA, USA | Cat# 3513 |
| Multiwell 6 Well | FALCON, Lincoln Park, NJ, USA | Cat# 353046 |
| Corning 60mm × 15mm Style | Corning Inc., Corning, NY, USA | Cat# 430166 |
| Corning 100mm × 20mm Style Dish Cell Culture | Corning Inc., Corning, NY, USA | Cat# 430167 |
| Corning 175cm2 Cell Culture Flask | Corning Inc., Corning, NY, USA | Cat# 431080 |
| Corning 500mL Bottle Top Vacuum Filter with 0.22µm Cellulose Acetate Membrane | Corning Inc., Corning, NY, USA | Cat# 430513 |
| poly-L-lysine coated glass-bottom dish | Matsunami, Osaka, Japan | Cat# D11141H |
| DNP-MRI system | JAPAN Redox Inc. ,Fukuoka, Japan |  |
| MC-PROXYL | JAPAN Redox Inc. ,Fukuoka, Japan |  |
| Scanning electron microscopy | Hitachi, Tokyo, Japan | Cat# S-4800 |
| Seahorse XFp Cell Culture Miniplate | Agilent Technologies, Santa Clara, CA, USA | Ca# 103025-100 |

| **Supplementary Table S2: 303 GO gene sets analysed by GSEA increased in IG27 cells versus control WT cells** | | | | |
| --- | --- | --- | --- | --- |
| NAME | SIZE* | NES# | NOM p-value # | FDR q-value# |
| GO_HEXOSE_METABOLIC_PROCESS | 84 | 1.879751 | 0 | 0.1442409 |
| GO_POSITIVE_REGULATION_OF_BIOMINERAL_TISSUE_DEVELOPMENT | 17 | 1.885686 | 0 | 0.147987 |
| GO_MONOSACCHARIDE_BIOSYNTHETIC_PROCESS | 31 | 1.8903217 | 0 | 0.1524142 |
| GO_OXIDOREDUCTASE_ACTIVITY_ACTING_ON_PAIRED_DONORS_WITH_INCORPORATION_OR_REDUCTION_OF_MOLECULAR_OXYGEN_2_OXOGLUTARATE_AS_ONE_DONOR_AND_INCORPORATION_OF_ONE_ATOM_EACH_OF_OXYGEN_INTO_BOTH_DONORS | 16 | 1.9032245 | 0 | 0.1542399 |
| GO_CELLULAR_CARBOHYDRATE_METABOLIC_PROCESS | 71 | 1.9169104 | 0 | 0.1566811 |
| GO_POSITIVE_REGULATION_OF_RESPONSE_TO_EXTRACELLULAR_STIMULUS | 20 | 1.9675639 | 0 | 0.1644252 |
| GO_SPROUTING_ANGIOGENESIS | 31 | 1.9246875 | 0 | 0.1692662 |
| GO_GLUCOSE_METABOLIC_PROCESS | 65 | 1.839148 | 0 | 0.1710759 |
| GO_NUCLEOSIDE_DIPHOSPHATE_METABOLIC_PROCESS | 42 | 1.8136557 | 0 | 0.172229 |
| GO_NUCLEOTIDE_PHOSPHORYLATION | 28 | 1.8143215 | 0 | 0.1775122 |
| GO_EUCHROMATIN | 18 | 1.8413374 | 0 | 0.177845 |
| GO_DEVELOPMENTAL_CELL_GROWTH | 32 | 1.8190403 | 0 | 0.1791759 |
| GO_MONOSACCHARIDE_CATABOLIC_PROCESS | 33 | 1.9301277 | 0 | 0.1792631 |
| GO_POLYSACCHARIDE_METABOLIC_PROCESS | 43 | 1.8259584 | 0 | 0.1795427 |
| GO_POSITIVE_REGULATION_OF_INTERFERON_BETA_PRODUCTION | 24 | 1.7862742 | 0 | 0.1800003 |
| GO_CELLULAR_RESPONSE_TO_NUTRIENT | 21 | 1.7793046 | 0 | 0.1806636 |
| GO_CELL_GROWTH | 60 | 1.991087 | 0 | 0.1807098 |
| GO_NADH_METABOLIC_PROCESS | 22 | 1.850815 | 0 | 0.1809084 |
| GO_NEGATIVE_REGULATION_OF_FAT_CELL_DIFFERENTIATION | 24 | 1.8536654 | 0 | 0.1819521 |
| GO_EMBRYONIC_EYE_MORPHOGENESIS | 20 | 1.7866613 | 0 | 0.1842763 |
| GO_RESPONSE_TO_ARSENIC_CONTAINING_SUBSTANCE | 22 | 1.8205028 | 0 | 0.1853347 |
| GO_AGING | 153 | 1.7934563 | 0 | 0.1859985 |
| GO_PROTEOGLYCAN_METABOLIC_PROCESS | 42 | 1.789859 | 0 | 0.1861334 |
| GO_HEXOSE_CATABOLIC_PROCESS | 27 | 1.7920371 | 0 | 0.1863642 |
| GO_BASAL_PLASMA_MEMBRANE | 19 | 1.7968104 | 0 | 0.1894671 |
| GO_REGULATION_OF_NUCLEOTIDE_CATABOLIC_PROCESS | 18 | 1.8554292 | 0 | 0.1909486 |
| GO_REGULATION_OF_SODIUM_ION_TRANSPORT | 38 | 1.8011135 | 0 | 0.1914212 |
| GO_POSITIVE_REGULATION_OF_REACTIVE_OXYGEN_SPECIES_BIOSYNTHETIC_PROCESS | 28 | 1.7633029 | 0 | 0.1957257 |
| GO_RESPONSE_TO_TYPE_I_INTERFERON | 30 | 1.7679982 | 0 | 0.196777 |
| GO_MONOSACCHARIDE_BINDING | 41 | 1.9313971 | 0 | 0.1998073 |
| GO_DIOXYGENASE_ACTIVITY | 33 | 2.0015137 | 0 | 0.2002913 |
| GO_RESPONSE_TO_OXYGEN_LEVELS | 198 | 1.7553502 | 0 | 0.2069812 |
| GO_PLASMA_MEMBRANE_RAFT | 53 | 1.5982193 | 0 | 0.2234079 |
| GO_POSITIVE_REGULATION_OF_INTRINSIC_APOPTOTIC_SIGNALING_PATHWAY | 27 | 1.598615 | 0 | 0.2239085 |
| GO_SULFOTRANSFERASE_ACTIVITY | 23 | 1.597308 | 0 | 0.224664 |
| GO_REGULATION_OF_MULTICELLULAR_ORGANISMAL_METABOLIC_PROCESS | 20 | 1.5986735 | 0 | 0.2249981 |
| GO_CELLULAR_RESPONSE_TO_AMINO_ACID_STIMULUS | 31 | 1.7349471 | 0 | 0.2253427 |
| GO_IMPORT_INTO_CELL | 21 | 1.5962731 | 0 | 0.2257828 |
| GO_TRANSFERASE_COMPLEX_TRANSFERRING_PHOSPHORUS_CONTAINING_GROUPS | 117 | 1.7463809 | 0 | 0.2259335 |
| GO_ANTIGEN_BINDING | 28 | 1.5993402 | 0 | 0.2260429 |
| GO_LIPOPOLYSACCHARIDE_MEDIATED_SIGNALING_PATHWAY | 21 | 1.7381998 | 0 | 0.2263815 |
| GO_ORGANELLE_DISASSEMBLY | 79 | 1.5997701 | 0 | 0.2264442 |
| GO_CELLULAR_RESPONSE_TO_DRUG | 46 | 1.568552 | 0 | 0.2266914 |
| GO_REGULATION_OF_MEMBRANE_REPOLARIZATION | 21 | 1.555063 | 0 | 0.2267844 |
| GO_HEPATICOBILIARY_SYSTEM_DEVELOPMENT | 76 | 1.5559578 | 0 | 0.2268781 |
| GO_COLUMNAR_CUBOIDAL_EPITHELIAL_CELL_DEVELOPMENT | 29 | 1.5545746 | 0 | 0.226952 |
| GO_CHEMOKINE_RECEPTOR_BINDING | 31 | 1.567747 | 0 | 0.2272728 |
| GO_TRANSCRIPTION_FROM_RNA_POLYMERASE_I_PROMOTER | 24 | 1.7139784 | 0 | 0.2273353 |
| GO_SKELETAL_MUSCLE_CELL_DIFFERENTIATION | 33 | 1.5633144 | 0 | 0.2273978 |
| GO_RNA_POLYMERASE_II_TRANSCRIPTION_FACTOR_BINDING | 63 | 1.6001499 | 0 | 0.2275525 |
| GO_ACTIN_CYTOSKELETON | 225 | 1.5563439 | 0 | 0.2276072 |
| GO_SODIUM_CHANNEL_REGULATOR_ACTIVITY | 16 | 1.6365688 | 0 | 0.2277355 |
| GO_POSITIVE_REGULATION_OF_EPITHELIAL_CELL_DIFFERENTIATION | 35 | 1.5686202 | 0 | 0.2277716 |
| GO_UROGENITAL_SYSTEM_DEVELOPMENT | 177 | 1.552834 | 0 | 0.2278071 |
| GO_CELLULAR_RESPONSE_TO_RETINOIC_ACID | 40 | 1.6011502 | 0 | 0.2278479 |
| GO_NEUROMUSCULAR_PROCESS | 55 | 1.5566856 | 0 | 0.2279267 |
| GO_INNATE_IMMUNE_RESPONSE | 256 | 1.5621636 | 0 | 0.2279822 |
| GO_REGULATION_OF_LEUKOCYTE_APOPTOTIC_PROCESS | 44 | 1.5635566 | 0 | 0.2279851 |
| GO_ENDODERM_DEVELOPMENT | 44 | 1.5575694 | 0 | 0.2280209 |
| GO_RESPONSE_TO_AMINO_ACID | 66 | 1.5651447 | 0 | 0.2280799 |
| GO_REGULATION_OF_ODONTOGENESIS | 16 | 1.5607874 | 0 | 0.2281469 |
| GO_PYRUVATE_METABOLIC_PROCESS | 36 | 1.5598344 | 0 | 0.2281924 |
| GO_POSITIVE_REGULATION_OF_SMOOTH_MUSCLE_CELL_MIGRATION | 24 | 1.5583186 | 0 | 0.2284189 |
| GO_RESPONSE_TO_X_RAY | 19 | 1.5603004 | 0 | 0.2285014 |
| GO_PROTEIN_KINASE_B_SIGNALING | 24 | 1.6399939 | 0 | 0.2285872 |
| GO_CELLULAR_RESPONSE_TO_ACID_CHEMICAL | 104 | 1.5528433 | 0 | 0.2287668 |
| GO_E_BOX_BINDING | 21 | 1.6426151 | 0 | 0.2288107 |
| GO_POSITIVE_REGULATION_OF_REACTIVE_OXYGEN_SPECIES_METABOLIC_PROCESS | 51 | 1.5687841 | 0 | 0.2288658 |
| GO_REGULATION_OF_INTERFERON_BETA_PRODUCTION | 31 | 1.5610621 | 0 | 0.228923 |
| GO_REGULATION_OF_REACTIVE_OXYGEN_SPECIES_BIOSYNTHETIC_PROCESS | 38 | 1.7401226 | 0 | 0.228925 |
| GO_DEFENSE_RESPONSE_TO_GRAM_NEGATIVE_BACTERIUM | 24 | 1.6016834 | 0 | 0.2289504 |
| GO_GLUCAN_METABOLIC_PROCESS | 30 | 1.7156355 | 0 | 0.2293504 |
| GO_EXTRACELLULAR_MATRIX_COMPONENT | 76 | 1.5704122 | 0 | 0.2293554 |
| GO_POLYSACCHARIDE_BIOSYNTHETIC_PROCESS | 22 | 1.5583661 | 0 | 0.2294272 |
| GO_CYCLIN_DEPENDENT_PROTEIN_KINASE_HOLOENZYME_COMPLEX | 15 | 1.5611414 | 0 | 0.2294385 |
| GO_REGULATION_OF_HAIR_CYCLE | 16 | 1.5591048 | 0 | 0.2294796 |
| GO_REGULATION_OF_DNA_TEMPLATED_TRANSCRIPTION_INITIATION | 17 | 1.6381035 | 0 | 0.2295636 |
| GO_REGULATION_OF_CARDIAC_MUSCLE_CONTRACTION | 44 | 1.57634 | 0 | 0.2295846 |
| GO_CELLULAR_RESPONSE_TO_EXTRACELLULAR_STIMULUS | 101 | 1.6369635 | 0 | 0.2296226 |
| GO_NEGATIVE_REGULATION_OF_REPRODUCTIVE_PROCESS | 31 | 1.5691353 | 0 | 0.2296964 |
| GO_PROTEIN_KINASE_COMPLEX | 48 | 1.7413218 | 0 | 0.2300649 |
| GO_REGULATION_OF_TRANSCRIPTION_REGULATORY_REGION_DNA_BINDING | 23 | 1.6019131 | 0 | 0.2303797 |
| GO_INTRAMOLECULAR_TRANSFERASE_ACTIVITY | 15 | 1.5750383 | 0 | 0.2304263 |
| GO_REGULATION_OF_ISOTYPE_SWITCHING | 15 | 1.5909629 | 0 | 0.2304411 |
| GO_REGULATION_OF_SYSTEMIC_ARTERIAL_BLOOD_PRESSURE_MEDIATED_BY_A_CHEMICAL_SIGNAL | 22 | 1.6429743 | 0 | 0.2304767 |
| GO_RESPONSE_TO_STEROL | 17 | 1.5704417 | 0 | 0.2304811 |
| GO_POSITIVE_REGULATION_OF_RELEASE_OF_CYTOCHROME_C_FROM_MITOCHONDRIA | 16 | 1.6404488 | 0 | 0.2305488 |
| GO_POSITIVE_REGULATION_OF_LYMPHOCYTE_MIGRATION | 19 | 1.5913328 | 0 | 0.2306861 |
| GO_OXIDOREDUCTASE_ACTIVITY | 314 | 1.577007 | 0 | 0.2307654 |
| GO_REGULATION_OF_COFACTOR_METABOLIC_PROCESS | 24 | 1.5705305 | 0 | 0.2310472 |
| GO_ADP_METABOLIC_PROCESS | 24 | 1.572797 | 0 | 0.2311703 |
| GO_DEVELOPMENTAL_GROWTH_INVOLVED_IN_MORPHOGENESIS | 52 | 1.7161038 | 0 | 0.2315041 |
| GO_NAD_METABOLIC_PROCESS | 28 | 1.601929 | 0 | 0.2318328 |
| GO_AMINOGLYCAN_BIOSYNTHETIC_PROCESS | 63 | 1.5710069 | 0 | 0.2318481 |
| GO_POSITIVE_REGULATION_OF_PROTEIN_TYROSINE_KINASE_ACTIVITY | 22 | 1.5770545 | 0 | 0.2319627 |
| GO_CELLULAR_RESPONSE_TO_EXTERNAL_STIMULUS | 151 | 1.5773789 | 0 | 0.2319672 |
| GO_REGULATION_OF_THE_FORCE_OF_HEART_CONTRACTION | 19 | 1.5914758 | 0 | 0.2320094 |
| GO_REGULATION_OF_KIDNEY_DEVELOPMENT | 31 | 1.572065 | 0 | 0.2320326 |
| GO_REGULATION_OF_SYSTEMIC_ARTERIAL_BLOOD_PRESSURE_BY_HORMONE | 17 | 1.5491104 | 0 | 0.2321025 |
| GO_REGULATION_OF_PEPTIDASE_ACTIVITY | 212 | 1.5457473 | 0 | 0.2321763 |
| GO_MACROAUTOPHAGY | 112 | 1.6266615 | 0 | 0.2323194 |
| GO_POTASSIUM_ION_IMPORT | 16 | 1.5890387 | 0 | 0.2323648 |
| GO_CELLULAR_SENESCENCE | 17 | 1.6255187 | 0 | 0.2324799 |
| GO_POSITIVE_REGULATION_OF_CHEMOKINE_PRODUCTION | 31 | 1.6437165 | 0 | 0.2325293 |
| GO_VITAMIN_TRANSPORT | 19 | 1.5459963 | 0 | 0.232568 |
| GO_EXTRACELLULAR_STRUCTURE_ORGANIZATION | 187 | 1.547607 | 0 | 0.2326259 |
| GO_CELL_SUBSTRATE_JUNCTION | 214 | 1.5834098 | 0 | 0.2326592 |
| GO_MONOCYTE_CHEMOTAXIS | 22 | 1.6022445 | 0 | 0.2326827 |
| GO_EAR_DEVELOPMENT | 123 | 1.5472728 | 0 | 0.2327181 |
| GO_CELLULAR_RESPONSE_TO_VASCULAR_ENDOTHELIAL_GROWTH_FACTOR_STIMULUS | 16 | 1.7205436 | 0 | 0.2328851 |
| GO_BASEMENT_MEMBRANE | 56 | 1.5774266 | 0 | 0.2331892 |
| GO_NEGATIVE_REGULATION_OF_LEUKOCYTE_MIGRATION | 21 | 1.616062 | 0 | 0.2333745 |
| GO_REGULATION_OF_CARDIAC_MUSCLE_CELL_MEMBRANE_REPOLARIZATION | 15 | 1.6271755 | 0 | 0.2334141 |
| GO_RESPONSE_TO_NICOTINE | 33 | 1.5792328 | 0 | 0.2334584 |
| GO_POSITIVE_REGULATION_OF_PROTEIN_COMPLEX_ASSEMBLY | 109 | 1.5779495 | 0 | 0.2337387 |
| GO_RESPONSE_TO_RETINOIC_ACID | 66 | 1.61684 | 0 | 0.2338066 |
| GO_RIBONUCLEOSIDE_DIPHOSPHATE_METABOLIC_PROCESS | 33 | 1.628197 | 0 | 0.2338885 |
| GO_BLOOD_VESSEL_ENDOTHELIAL_CELL_MIGRATION | 19 | 1.5837039 | 0 | 0.23393 |
| GO_RESPONSE_TO_INTERLEUKIN_1 | 68 | 1.617379 | 0 | 0.2340324 |
| GO_ENDOCRINE_PROCESS | 22 | 1.6023924 | 0 | 0.2341799 |
| GO_RESPONSE_TO_VIRUS | 118 | 1.5858647 | 0 | 0.2341886 |
| GO_REGULATION_OF_SODIUM_ION_TRANSMEMBRANE_TRANSPORT | 26 | 1.614639 | 0 | 0.2342461 |
| GO_BASAL_PART_OF_CELL | 31 | 1.6200217 | 0 | 0.2342506 |
| GO_MACROPHAGE_ACTIVATION | 18 | 1.6775765 | 0 | 0.2342884 |
| GO_REGULATION_OF_ENDOPLASMIC_RETICULUM_STRESS_INDUCED_INTRINSIC_APOPTOTIC_SIGNALING_PATHWAY | 17 | 1.6095748 | 0 | 0.2343427 |
| GO_CELLULAR_RESPONSE_TO_ALCOHOL | 64 | 1.5852501 | 0 | 0.2344072 |
| GO_POSITIVE_REGULATION_OF_POTASSIUM_ION_TRANSMEMBRANE_TRANSPORT | 19 | 1.629933 | 0 | 0.2344123 |
| GO_NEGATIVE_REGULATION_OF_CATION_TRANSMEMBRANE_TRANSPORT | 46 | 1.579814 | 0 | 0.2344613 |
| GO_CELLULAR_RESPONSE_TO_AMINO_ACID_STARVATION | 16 | 1.6206573 | 0 | 0.2346112 |
| GO_NEGATIVE_REGULATION_OF_I_KAPPAB_KINASE_NF_KAPPAB_SIGNALING | 21 | 1.6438235 | 0 | 0.2346307 |
| GO_ATP_GENERATION_FROM_ADP | 20 | 1.5433095 | 0 | 0.2346964 |
| GO_ENERGY_RESERVE_METABOLIC_PROCESS | 35 | 1.7079326 | 0 | 0.2347659 |
| GO_REGULATION_OF_CARBOHYDRATE_CATABOLIC_PROCESS | 20 | 1.6154287 | 0 | 0.2349421 |
| GO_MYELOID_LEUKOCYTE_ACTIVATION | 59 | 1.6755615 | 0 | 0.2351645 |
| GO_RAC_GTPASE_BINDING | 20 | 1.5838556 | 0 | 0.2352193 |
| GO_POSITIVE_REGULATION_OF_KIDNEY_DEVELOPMENT | 24 | 1.6027598 | 0.071 | 0.2353328 |
| GO_CELLULAR_RESPONSE_TO_OXYGEN_LEVELS | 77 | 1.630488 | 0 | 0.2355271 |
| GO_RESPONSE_TO_INTERFERON_GAMMA | 68 | 1.6102654 | 0 | 0.2355484 |
| GO_CELLULAR_CARBOHYDRATE_BIOSYNTHETIC_PROCESS | 23 | 1.7234151 | 0 | 0.2356246 |
| GO_CELLULAR_RESPONSE_TO_OXIDATIVE_STRESS | 111 | 1.5433408 | 0 | 0.2356571 |
| GO_DNA_PACKAGING | 66 | 1.6810001 | 0 | 0.235674 |
| GO_CHROMATIN_SILENCING | 28 | 1.6174349 | 0 | 0.235728 |
| GO_ORGAN_REGENERATION | 50 | 1.6444563 | 0 | 0.2357398 |
| GO_ANCHORING_JUNCTION | 256 | 1.6283017 | 0 | 0.2357609 |
| GO_NEGATIVE_REGULATION_OF_TUMOR_NECROSIS_FACTOR_SUPERFAMILY_CYTOKINE_PRODUCTION | 19 | 1.6036803 | 0 | 0.2358004 |
| GO_REGULATION_OF_SODIUM_ION_TRANSMEMBRANE_TRANSPORTER_ACTIVITY | 23 | 1.7044257 | 0 | 0.2358082 |
| GO_CARDIAC_MUSCLE_CELL_ACTION_POTENTIAL | 32 | 1.6211411 | 0 | 0.2360097 |
| GO_EMBRYONIC_CAMERA_TYPE_EYE_MORPHOGENESIS | 15 | 1.7210736 | 0 | 0.2360658 |
| GO_REGULATION_OF_LYMPHOCYTE_MIGRATION | 26 | 1.6218883 | 0 | 0.2361642 |
| GO_CELLULAR_RESPONSE_TO_BIOTIC_STIMULUS | 93 | 1.667282 | 0 | 0.2362861 |
| GO_EXTRACELLULAR_MATRIX_BINDING | 34 | 1.6729842 | 0 | 0.2363304 |
| GO_LUNG_MORPHOGENESIS | 26 | 1.6176612 | 0 | 0.2363408 |
| GO_HETEROTYPIC_CELL_CELL_ADHESION | 17 | 1.5392121 | 0 | 0.2363755 |
| GO_PURINERGIC_RECEPTOR_SIGNALING_PATHWAY | 17 | 1.5387094 | 0 | 0.2364216 |
| GO_STEM_CELL_DIFFERENTIATION | 103 | 1.6108488 | 0 | 0.2364514 |
| GO_CARBOHYDRATE_CATABOLIC_PROCESS | 63 | 1.6781965 | 0 | 0.2365016 |
| GO_NUCLEAR_CHROMATIN | 144 | 1.540284 | 0 | 0.2366434 |
| GO_GLANDULAR_EPITHELIAL_CELL_DIFFERENTIATION | 18 | 1.6052755 | 0 | 0.2366803 |
| GO_NEGATIVE_REGULATION_OF_ERK1_AND_ERK2_CASCADE | 28 | 1.6598456 | 0 | 0.2370491 |
| GO_REGULATION_OF_DNA_TEMPLATED_TRANSCRIPTION_IN_RESPONSE_TO_STRESS | 33 | 1.6604533 | 0 | 0.2372759 |
| GO_REGULATION_OF_VASODILATION | 29 | 1.6068307 | 0 | 0.2373006 |
| GO_NEURON_PROJECTION_EXTENSION | 24 | 1.5373847 | 0 | 0.2373231 |
| GO_CELLULAR_RESPONSE_TO_CALCIUM_ION | 26 | 1.6499575 | 0 | 0.2375419 |
| GO_PEPTIDYL_PROLINE_MODIFICATION | 35 | 1.5414115 | 0 | 0.23759 |
| GO_PHOSPHATIDYLCHOLINE_METABOLIC_PROCESS | 24 | 1.5402855 | 0 | 0.2375991 |
| GO_CHEMOKINE_ACTIVITY | 24 | 1.5370387 | 0 | 0.2376125 |
| GO_PALATE_DEVELOPMENT | 51 | 1.6528033 | 0 | 0.2376372 |
| GO_REGENERATION | 102 | 1.5326364 | 0 | 0.2377101 |
| GO_CELLULAR_EXTRAVASATION | 19 | 1.5337764 | 0 | 0.2378015 |
| GO_NEGATIVE_REGULATION_OF_RESPONSE_TO_ENDOPLASMIC_RETICULUM_STRESS | 19 | 1.6454674 | 0 | 0.2379318 |
| GO_AXON_EXTENSION | 17 | 1.6465626 | 0 | 0.2379621 |
| GO_RESPONSE_TO_ENDOPLASMIC_RETICULUM_STRESS | 115 | 1.5357628 | 0 | 0.2380219 |
| GO_SMALL_MOLECULE_BIOSYNTHETIC_PROCESS | 217 | 1.5260143 | 0 | 0.2380718 |
| GO_RESPONSE_TO_STARVATION | 86 | 1.5332438 | 0 | 0.2381 |
| GO_REGULATION_OF_ACTIN_CYTOSKELETON_REORGANIZATION | 20 | 1.6646658 | 0 | 0.2381435 |
| GO_CHROMATIN_ASSEMBLY_OR_DISASSEMBLY | 60 | 1.5349917 | 0 | 0.238197 |
| GO_GLUCOSE_CATABOLIC_PROCESS | 16 | 1.7239976 | 0 | 0.2382087 |
| GO_REGULATION_OF_T_CELL_MIGRATION | 17 | 1.5378879 | 0 | 0.2382625 |
| GO_MULTICELLULAR_ORGANISM_METABOLIC_PROCESS | 59 | 1.5342822 | 0 | 0.2383096 |
| GO_MOTOR_NEURON_AXON_GUIDANCE | 17 | 1.5371083 | 0 | 0.2383589 |
| GO_OXIDATION_REDUCTION_PROCESS | 408 | 1.6686412 | 0 | 0.2384516 |
| GO_POSITIVE_REGULATION_OF_CELLULAR_COMPONENT_BIOGENESIS | 217 | 1.5261997 | 0 | 0.2386764 |
| GO_CELLULAR_RESPONSE_TO_VITAMIN | 15 | 1.6810043 | 0 | 0.2387195 |
| GO_HEPARAN_SULFATE_PROTEOGLYCAN_METABOLIC_PROCESS | 15 | 1.670316 | 0 | 0.2387535 |
| GO_POSITIVE_REGULATION_OF_ENDOCYTOSIS | 76 | 1.653603 | 0 | 0.2389047 |
| GO_RESPONSE_TO_MOLECULE_OF_BACTERIAL_ORIGIN | 181 | 1.5289689 | 0 | 0.239049 |
| GO_REGULATION_OF_RECEPTOR_MEDIATED_ENDOCYTOSIS | 49 | 1.5265949 | 0 | 0.2390804 |
| GO_REGULATION_OF_LIPASE_ACTIVITY | 51 | 1.5262953 | 0 | 0.2391385 |
| GO_NEGATIVE_REGULATION_OF_CYTOKINE_PRODUCTION | 110 | 1.6470426 | 0 | 0.2391548 |
| GO_CELLULAR_ALDEHYDE_METABOLIC_PROCESS | 38 | 1.6553658 | 0 | 0.2392428 |
| GO_IMMUNOLOGICAL_SYNAPSE | 19 | 1.5285445 | 0 | 0.2392466 |
| GO_OXIDOREDUCTION_COENZYME_METABOLIC_PROCESS | 49 | 1.5224857 | 0 | 0.2392577 |
| GO_REGULATION_OF_MYOBLAST_DIFFERENTIATION | 28 | 1.6617663 | 0 | 0.2394249 |
| GO_RESPONSE_TO_MECHANICAL_STIMULUS | 130 | 1.5271065 | 0 | 0.2395577 |
| GO_NEUROMUSCULAR_PROCESS_CONTROLLING_BALANCE | 30 | 1.5225463 | 0 | 0.2397012 |
| GO_RESPONSE_TO_PEPTIDE | 248 | 1.5293945 | 0 | 0.2397276 |
| GO_CARBOHYDRATE_BINDING | 137 | 1.6547902 | 0 | 0.2397606 |
| GO_POSITIVE_REGULATION_OF_TYPE_I_INTERFERON_PRODUCTION | 45 | 1.6822504 | 0 | 0.2398086 |
| GO_CORTICAL_ACTIN_CYTOSKELETON | 26 | 1.6505253 | 0 | 0.2398695 |
| GO_POSITIVE_REGULATION_OF_LEUKOCYTE_MIGRATION | 76 | 1.52997 | 0 | 0.2402685 |
| GO_NEGATIVE_REGULATION_OF_RESPONSE_TO_EXTERNAL_STIMULUS | 146 | 1.5271398 | 0 | 0.2402946 |
| GO_MULTICELLULAR_ORGANISM_AGING | 16 | 1.6561284 | 0 | 0.2405851 |
| GO_POSITIVE_REGULATION_OF_ADAPTIVE_IMMUNE_RESPONSE | 48 | 1.5302678 | 0 | 0.2407866 |
| GO_POSITIVE_REGULATION_OF_EMBRYONIC_DEVELOPMENT | 16 | 1.5307183 | 0 | 0.2409715 |
| GO_PROTEOGLYCAN_BIOSYNTHETIC_PROCESS | 27 | 1.6507474 | 0 | 0.2418289 |
| GO_CELL_AGING | 33 | 1.699209 | 0 | 0.2420696 |
| GO_POSITIVE_REGULATION_OF_RECEPTOR_MEDIATED_ENDOCYTOSIS | 30 | 1.70037 | 0 | 0.2427538 |
| GO_INTRINSIC_APOPTOTIC_SIGNALING_PATHWAY_IN_RESPONSE_TO_DNA_DAMAGE_BY_P53_CLASS_MEDIATOR | 17 | 1.682493 | 0 | 0.2430332 |
| GO_GROWTH | 216 | 1.5117322 | 0 | 0.2444134 |
| GO_EPITHELIAL_CELL_DIFFERENTIATION_INVOLVED_IN_KIDNEY_DEVELOPMENT | 15 | 1.5066962 | 0 | 0.2445084 |
| GO_PROTEIN_HETERODIMERIZATION_ACTIVITY | 219 | 1.5079886 | 0 | 0.2447181 |
| GO_NEGATIVE_REGULATION_OF_INTRACELLULAR_PROTEIN_TRANSPORT | 48 | 1.5082461 | 0 | 0.2447725 |
| GO_EAR_MORPHOGENESIS | 70 | 1.5072614 | 0 | 0.244806 |
| GO_CELLULAR_RESPONSE_TO_INTERLEUKIN_1 | 52 | 1.511767 | 0 | 0.2450443 |
| GO_FACE_DEVELOPMENT | 32 | 1.4616653 | 0 | 0.2452451 |
| GO_POSITIVE_REGULATION_OF_CYTOKINE_PRODUCTION_INVOLVED_IN_IMMUNE_RESPONSE | 19 | 1.5187863 | 0 | 0.2453291 |
| GO_CELL_CYCLE_ARREST | 79 | 1.5084147 | 0 | 0.2453867 |
| GO_POSITIVE_REGULATION_OF_RESPONSE_TO_EXTERNAL_STIMULUS | 162 | 1.5057076 | 0 | 0.2454105 |
| GO_MULTICELLULAR_ORGANISMAL_MACROMOLECULE_METABOLIC_PROCESS | 52 | 1.4596381 | 0 | 0.2454619 |
| GO_POSITIVE_REGULATION_OF_PATHWAY_RESTRICTED_SMAD_PROTEIN_PHOSPHORYLATION | 32 | 1.5122137 | 0 | 0.2455476 |
| GO_MYELOID_DENDRITIC_CELL_ACTIVATION | 16 | 1.5112987 | 0 | 0.245604 |
| GO_AMMONIUM_TRANSMEMBRANE_TRANSPORTER_ACTIVITY | 17 | 1.6826204 | 0 | 0.2457426 |
| GO_TUBE_FORMATION | 66 | 1.459916 | 0 | 0.2458116 |
| GO_METHYLATION | 104 | 1.4616829 | 0 | 0.2458239 |
| GO_RESPONSE_TO_CORTICOSTEROID | 95 | 1.5107175 | 0 | 0.245905 |
| GO_POSITIVE_REGULATION_OF_RESPONSE_TO_WOUNDING | 94 | 1.5085391 | 0 | 0.2461891 |
| GO_REGULATION_OF_PROTEIN_DEACETYLATION | 15 | 1.6848392 | 0 | 0.2462639 |
| GO_CELLULAR_RESPONSE_TO_OXYGEN_CONTAINING_COMPOUND | 469 | 1.4600811 | 0 | 0.2462702 |
| GO_POSITIVE_REGULATION_OF_MYELOID_CELL_DIFFERENTIATION | 45 | 1.460329 | 0 | 0.2462977 |
| GO_SERINE_TYPE_ENDOPEPTIDASE_INHIBITOR_ACTIVITY | 49 | 1.4617542 | 0 | 0.2463049 |
| GO_RIBONUCLEOPROTEIN_COMPLEX_LOCALIZATION | 48 | 1.5122377 | 0 | 0.2463789 |
| GO_MACROMITOPHAGY | 59 | 1.5088512 | 0 | 0.2464861 |
| GO_MONOSACCHARIDE_TRANSPORT | 31 | 1.5019097 | 0 | 0.2465911 |
| GO_REGULATION_OF_PHOSPHOLIPASE_ACTIVITY | 41 | 1.4621521 | 0 | 0.2466201 |
| GO_ION_GATED_CHANNEL_ACTIVITY | 15 | 1.4994177 | 0.096 | 0.2466809 |
| GO_MESENCHYMAL_CELL_DIFFERENTIATION | 74 | 1.496711 | 0 | 0.2466961 |
| GO_PROTEIN_ALKYLATION | 40 | 1.4961741 | 0 | 0.2467018 |
| GO_RESPONSE_TO_VITAMIN_D | 18 | 1.6930794 | 0 | 0.2467476 |
| GO_MORPHOGENESIS_OF_AN_EPITHELIAL_SHEET | 26 | 1.4979064 | 0 | 0.2468753 |
| GO_CARBOHYDRATE_DERIVATIVE_BIOSYNTHETIC_PROCESS | 315 | 1.4623063 | 0 | 0.2469065 |
| GO_REGULATION_OF_CELL_MATRIX_ADHESION | 65 | 1.5123467 | 0 | 0.2469066 |
| GO_HISTONE_ACETYLTRANSFERASE_BINDING | 18 | 1.4934452 | 0 | 0.246983 |
| GO_RNA_CAPPING | 21 | 1.4985447 | 0.084 | 0.247031 |
| GO_DNA_PACKAGING_COMPLEX | 26 | 1.513091 | 0 | 0.247206 |
| GO_CELLULAR_KETONE_METABOLIC_PROCESS | 32 | 1.4995366 | 0 | 0.2472635 |
| GO_RESPONSE_TO_CYTOKINE | 389 | 1.5088888 | 0 | 0.2473002 |
| GO_GLYCEROLIPID_CATABOLIC_PROCESS | 17 | 1.5174005 | 0 | 0.2473078 |
| GO_PROTEIN_DNA_COMPLEX | 64 | 1.5019977 | 0 | 0.2473596 |
| GO_CARDIOCYTE_DIFFERENTIATION | 54 | 1.5042278 | 0 | 0.2473742 |
| GO_POSITIVE_REGULATION_OF_DENDRITE_MORPHOGENESIS | 17 | 1.5004133 | 0 | 0.2474131 |
| GO_SULFUR_COMPOUND_CATABOLIC_PROCESS | 26 | 1.4943292 | 0 | 0.2474283 |
| GO_GRANULOCYTE_MIGRATION | 43 | 1.4624082 | 0 | 0.247498 |
| GO_RESPONSE_TO_BIOTIC_STIMULUS | 413 | 1.4986385 | 0 | 0.2475483 |
| GO_GENITALIA_DEVELOPMENT | 28 | 1.502324 | 0 | 0.2475561 |
| GO_MUSCLE_ORGAN_DEVELOPMENT | 163 | 1.4670495 | 0 | 0.2475944 |
| GO_HEAD_MORPHOGENESIS | 23 | 1.4935663 | 0.082 | 0.2477064 |
| GO_ANGIOGENESIS | 178 | 1.5025374 | 0 | 0.2477068 |
| GO_DNA_TEMPLATED_TRANSCRIPTION_INITIATION | 105 | 1.5039917 | 0 | 0.2477421 |
| GO_EXTRACELLULAR_MATRIX | 253 | 1.462808 | 0 | 0.2477446 |
| GO_REGULATION_OF_NUCLEOSIDE_METABOLIC_PROCESS | 23 | 1.4663832 | 0 | 0.2478401 |
| GO_REGULATION_OF_RESPONSE_TO_CYTOKINE_STIMULUS | 76 | 1.5006869 | 0 | 0.247889 |
| GO_INSULIN_LIKE_GROWTH_FACTOR_BINDING | 15 | 1.4571214 | 0 | 0.2479487 |
| GO_POSITIVE_REGULATION_OF_AUTOPHAGY | 35 | 1.4995587 | 0 | 0.2480224 |
| GO_CORE_PROMOTER_SEQUENCE_SPECIFIC_DNA_BINDING | 54 | 1.4628768 | 0 | 0.2480264 |
| GO_SUGAR_TRANSMEMBRANE_TRANSPORTER_ACTIVITY | 17 | 1.4952627 | 0 | 0.2480421 |
| GO_CELLULAR_RESPONSE_TO_IONIZING_RADIATION | 26 | 1.4943401 | 0 | 0.2480461 |
| GO_PROTEIN_IMPORT_INTO_NUCLEUS_TRANSLOCATION | 19 | 1.5131699 | 0 | 0.2480558 |
| GO_AMINOGLYCAN_METABOLIC_PROCESS | 96 | 1.467134 | 0 | 0.248106 |
| GO_LIPID_TRANSLOCATION | 15 | 1.4664218 | 0 | 0.248455 |
| GO_INCLUSION_BODY | 36 | 1.4945523 | 0.091 | 0.2484811 |
| GO_SMALL_MOLECULE_CATABOLIC_PROCESS | 171 | 1.5027183 | 0 | 0.2484892 |
| GO_REGULATION_OF_HEART_RATE_BY_CARDIAC_CONDUCTION | 24 | 1.5136237 | 0 | 0.2485529 |
| GO_REGULATION_OF_CYTOKINE_PRODUCTION | 313 | 1.4630655 | 0 | 0.2486264 |
| GO_REGULATION_OF_CHEMOKINE_PRODUCTION | 41 | 1.6863244 | 0 | 0.2486294 |
| GO_RESPONSE_TO_WOUNDING | 346 | 1.4557322 | 0 | 0.2487038 |
| GO_GASTRULATION_WITH_MOUTH_FORMING_SECOND | 15 | 1.464026 | 0.079 | 0.2487073 |
| GO_RESPONSE_TO_ACTIVITY | 43 | 1.4562178 | 0 | 0.2487217 |
| GO_REGULATION_OF_PEPTIDYL_THREONINE_PHOSPHORYLATION | 22 | 1.4672129 | 0 | 0.2487277 |
| GO_EMBRYONIC_FORELIMB_MORPHOGENESIS | 15 | 1.4654324 | 0 | 0.2487637 |
| GO_REGULATION_OF_MORPHOGENESIS_OF_A_BRANCHING_STRUCTURE | 33 | 1.4708973 | 0 | 0.248909 |
| GO_GOLGI_LUMEN | 49 | 1.4674662 | 0 | 0.2489631 |
| GO_REGULATION_OF_OSSIFICATION | 99 | 1.4633738 | 0 | 0.2490019 |
| GO_PEPTIDASE_INHIBITOR_ACTIVITY | 93 | 1.4657608 | 0 | 0.2490383 |
| GO_MACROMOLECULE_METHYLATION | 76 | 1.4642974 | 0 | 0.249086 |
| GO_BASOLATERAL_PLASMA_MEMBRANE | 120 | 1.4702412 | 0 | 0.249169 |
| GO_AXIS_ELONGATION | 18 | 1.6887653 | 0 | 0.249191 |
| GO_REGULATION_OF_LEUKOCYTE_MIGRATION | 103 | 1.4646125 | 0 | 0.2491936 |
| GO_G_PROTEIN_COUPLED_RECEPTOR_BINDING | 140 | 1.4710121 | 0 | 0.2492028 |
| GO_ENZYME_INHIBITOR_ACTIVITY | 185 | 1.5136929 | 0 | 0.2492492 |
| GO_CARDIAC_CELL_DEVELOPMENT | 27 | 1.4688545 | 0 | 0.2494106 |
| GO_LEUKOCYTE_CHEMOTAXIS | 64 | 1.469288 | 0 | 0.24943 |
| GO_CARBOHYDRATE_METABOLIC_PROCESS | 338 | 1.4770409 | 0 | 0.2494639 |
| GO_OXIDOREDUCTASE_ACTIVITY_ACTING_ON_CH_OH_GROUP_OF_DONORS | 60 | 1.483994 | 0.082 | 0.2495103 |
| GO_ACTIN_FILAMENT_ORGANIZATION | 91 | 1.4751434 | 0 | 0.2495149 |
| GO_DNA_CONFORMATION_CHANGE | 113 | 1.4842342 | 0 | 0.249542 |
| GO_TRNA_MODIFICATION | 20 | 1.4542503 | 0 | 0.2495855 |
| GO_BLOOD_VESSEL_MORPHOGENESIS | 221 | 1.4674915 | 0 | 0.2495916 |
| GO_NEGATIVE_REGULATION_OF_RESPONSE_TO_WOUNDING | 93 | 1.4886636 | 0 | 0.2497018 |
| GO_KINASE_INHIBITOR_ACTIVITY | 44 | 1.4737406 | 0 | 0.2497382 |
| GO_CD4_POSITIVE_ALPHA_BETA_T_CELL_ACTIVATION | 20 | 1.4715593 | 0 | 0.2497465 |
| GO_RNA_POLYMERASE_COMPLEX | 57 | 1.4846772 | 0 | 0.249787 |
| GO_REGULATION_OF_CELLULAR_RESPONSE_TO_STRESS | 390 | 1.5159068 | 0 | 0.2498207 |
| GO_DNA_DIRECTED_RNA_POLYMERASE_II_HOLOENZYME | 39 | 1.4719293 | 0 | 0.2498475 |
| #NES, Normalized Enrichment Score; NOM, normal; FDR, False Discovery Rate | |  |  |  |
| *SIZE indicates number of genes in the gene set after filtering out those genes not in the expression dataset | | | |  |

| **Supplementary Table S3: metabolomics in IG 27cells versus Wt cells** | | | | | | | |  |  |  |
| --- | --- | --- | --- | --- | --- | --- | --- | --- | --- | --- |
|  | Concentration (pmol/10^6^ cells) | | | |  |  |  |  |  |  |
| Pathway Label | Control |  |  | Mutant |  |  | Control |  | Mutant |  |
|  | WT-1 | WT-2 | WT-3 | IG27-1 | IG27-2 | IG27-3 | Mean | S.D. | Mean | S.D. |
| NAD+ | 721 | 715 | 683 | 1,059 | 1,177 | 1,185 | 706 | 20 | 1,140 | 70 |
| cAMP | 1.9 | 1.7 | 1.5 | 1.5 | 2.0 | 1.7 | 1.7 | 0.2 | 1.7 | 0.2 |
| cGMP | N.D. | N.D. | N.D. | N.D. | N.D. | N.D. | N.A. | N.A. | N.A. | N.A. |
| NADH | 64 | 60 | 54 | 103 | 110 | 104 | 59 | 5.0 | 106 | 3.5 |
| Xanthine | 10 | 11 | 9.1 | 18 | 19 | 20 | 10 | 1.0 | 19 | 1.1 |
| ADP-Rib | 2.1 | 2.7 | 3.0 | 3.2 | 5.2 | 4.7 | 2.6 | 0.5 | 4.3 | 1.0 |
| Mevalonic acid | N.D. | N.D. | N.D. | N.D. | N.D. | N.D. | N.A. | N.A. | N.A. | N.A. |
| UDP-Glc | 811 | 788 | 775 | 1,450 | 1,601 | 1,672 | 791 | 18 | 1,574 | 114 |
| Uric acid | 11 | 15 | 11 | 16 | 14 | 17 | 13 | 2.1 | 15 | 1.5 |
| NADP+ | 50 | 48 | 47 | 71 | 99 | 104 | 48 | 1.5 | 92 | 18 |
| IMP | 71 | 63 | 58 | 151 | 171 | 157 | 64 | 6.4 | 160 | 10 |
| S7P | N.D. | N.D. | N.D. | N.D. | N.D. | N.D. | N.A. | N.A. | N.A. | N.A. |
| G6P | 62 | 69 | 66 | 70 | 80 | 92 | 66 | 3.9 | 80 | 11 |
| F6P | 23 | 22 | 23 | 24 | 28 | 30 | 23 | 0.4 | 27 | 3.3 |
| D-F1P | 51 | 22 | 28 | N.D. | 12 | N.D. | 34 | 15 | 12 | N.A. |
| Gal1P | 1.0 | N.D. | 1.5 | 5.5 | 5.8 | 3.2 | 1.3 | 0.3 | 4.8 | 1.4 |
| G1P | 12 | 12 | 10 | 15 | N.D. | 13 | 11 | 1.2 | 14 | 1.2 |
| AAcCoA | N.D. | N.D. | N.D. | N.D. | N.D. | N.D. | N.A. | N.A. | N.A. | N.A. |
| AcCoA | 15 | 11 | 5.7 | 26 | 39 | 47 | 10 | 4.4 | 38 | 11 |
| Folic acid | 1.2 | 1.3 | 1.1 | 2.6 | 2.9 | 3.2 | 1.2 | 0.07 | 2.9 | 0.3 |
| R5P | 14 | 11 | 26 | 36 | 41 | 60 | 17 | 7.9 | 46 | 13 |
| CoA | 25 | 26 | 32 | 35 | 36 | 37 | 28 | 3.5 | 36 | 0.9 |
| R1P | 19 | 18 | 18 | 22 | 20 | 18 | 18 | 0.2 | 20 | 2.1 |
| Ru5P | 15 | 8.7 | 21 | 42 | 38 | 55 | 15 | 6.0 | 45 | 9.1 |
| X5P | 24 | 20 | 40 | 53 | 101 | 99 | 28 | 11 | 84 | 27 |
| E4P | N.D. | N.D. | N.D. | 7.5 | 10 | 7.5 | N.A. | N.A. | 8.3 | 1.4 |
| HMG-CoA | 8.2 | 8.0 | 8.3 | 21 | 19 | 20 | 8.2 | 0.13 | 20 | 0.8 |
| Glyceraldehyde 3-phosphate | 121 | 125 | 157 | 61 | 73 | 58 | 134 | 19 | 64 | 7.8 |
| NADPH | 50 | 49 | 47 | 109 | 86 | 101 | 48 | 1.8 | 99 | 11 |
| Malonyl-CoA | 4.4 | 3.4 | 3.7 | 9.8 | 10 | 11 | 3.8 | 0.5 | 10 | 0.6 |
| Phosphocreatine | 8,321 | 8,143 | 7,804 | 7,822 | 7,562 | 7,363 | 8,089 | 263 | 7,582 | 231 |
| XMP | 2.0 | 1.8 | 1.7 | 3.2 | 3.3 | 3.3 | 1.8 | 0.14 | 3.3 | 0.05 |
| DHAP | 318 | 319 | 663 | 164 | 142 | 265 | 433 | 199 | 190 | 66 |
| Succinyl AMP | 4.1 | 3.8 | 3.4 | 5.3 | 6.0 | 5.3 | 3.8 | 0.3 | 5.5 | 0.4 |
| F1,6P | 164 | 134 | 143 | 234 | 270 | 260 | 147 | 15 | 255 | 19 |
| 6-PG | 27 | 24 | 25 | 21 | 23 | 24 | 25 | 1.5 | 23 | 1.9 |
| Carbamoyl-Asp | 71 | 68 | 68 | 118 | 121 | 127 | 69 | 1.7 | 122 | 4.7 |
| PRPP | 4.5 | 4.4 | 5.3 | 3.9 | 3.8 | 6.0 | 4.7 | 0.5 | 4.6 | 1.3 |
| 2-PG | 1.8 | 3.5 | 2.7 | 3.4 | 2.4 | 2.5 | 2.7 | 0.8 | 2.8 | 0.6 |
| Diphosphoglycerate | 113 | 102 | 111 | 40 | 36 | 60 | 109 | 5.9 | 45 | 13 |
| 3-PG | 25 | 31 | 29 | 31 | 26 | 23 | 28 | 2.7 | 26 | 4.0 |
| PEP | 6.5 | 8.0 | 6.9 | 8.8 | 8.2 | 7.1 | 7.1 | 0.7 | 8.0 | 0.9 |
| GMP | 10 | 11 | 10 | 18 | 7.5 | 8.1 | 10 | 0.2 | 11 | 5.7 |
| AMP | 47 | 58 | 39 | 100 | 52 | 49 | 48 | 9.8 | 67 | 28 |
| 2-KIV | 27 | 29 | 27 | 67 | 70 | 71 | 28 | 0.8 | 69 | 2.0 |
| GDP | 61 | 78 | 58 | 76 | 53 | 37 | 65 | 11 | 56 | 20 |
| Lactic acid | 8,962 | 8,008 | 7,799 | 20,937 | 25,242 | 23,350 | 8,256 | 620 | 23,176 | 2,158 |
| ADP | 264 | 341 | 238 | 218 | 189 | 292 | 281 | 54 | 233 | 53 |
| GTP | 1,283 | 1,173 | 1,157 | 1,552 | 1,900 | 2,103 | 1,204 | 68 | 1,852 | 279 |
| Glyoxylic acid | N.D. | N.D. | N.D. | N.D. | N.D. | N.D. | N.A. | N.A. | N.A. | N.A. |
| ATP | 7,901 | 7,228 | 6,913 | 11,354 | 13,236 | 13,751 | 7,348 | 505 | 12,780 | 1,262 |
| Glycerol 3-phosphate | 1,669 | 1,628 | 1,490 | 3,052 | 3,313 | 3,258 | 1,596 | 94 | 3,208 | 138 |
| Glycolic acid | N.D. | N.D. | N.D. | N.D. | N.D. | N.D. | N.A. | N.A. | N.A. | N.A. |
| Pyruvic acid | 767 | 768 | 718 | 705 | 800 | 858 | 751 | 29 | 788 | 77 |
| N-AcGlu | 13 | 12 | 11 | 15 | 18 | 18 | 12 | 0.7 | 17 | 1.8 |
| 2-Hydroxyglutaric acid | 64 | 63 | 50 | 150 | 180 | 193 | 59 | 7.6 | 174 | 22 |
| Carbamoyl-P | N.D. | N.D. | N.D. | N.D. | N.D. | N.D. | N.A. | N.A. | N.A. | N.A. |
| Succinic acid | 164 | 145 | 141 | 340 | 383 | 361 | 150 | 12 | 361 | 22 |
| Malic acid | 1,366 | 1,080 | 1,080 | 1,994 | 2,361 | 2,223 | 1,175 | 165 | 2,193 | 185 |
| 2-OG | 135 | 119 | 115 | 226 | 342 | 319 | 123 | 10 | 296 | 61 |
| Fumaric acid | 305 | 251 | 224 | 396 | 461 | 440 | 260 | 41 | 432 | 33 |
| Citric acid | 873 | 709 | 643 | 1,491 | 1,926 | 1,791 | 741 | 119 | 1,736 | 222 |
| cis-Aconitic acid | 13 | 13 | 12 | 31 | 40 | 39 | 12 | 0.7 | 37 | 5.0 |
| Isocitric acid | 39 | 35 | 31 | 83 | 109 | 109 | 35 | 4.2 | 100 | 15 |
| Urea | N.D. | N.D. | N.D. | N.D. | 1,691 | 1,441 | N.A. | N.A. | 1,566 | 176 |
| Gly | 25,332 | 26,169 | 25,550 | 72,213 | 89,526 | 83,459 | 25,684 | 434 | 81,733 | 8,785 |
| Putrescine | 229 | 259 | 243 | 189 | 293 | 294 | 244 | 15 | 259 | 60 |
| Ala | 52,770 | 51,654 | 52,811 | 62,763 | 75,058 | 69,916 | 52,412 | 657 | 69,246 | 6,175 |
| Sarcosine | N.D. | N.D. | N.D. | N.D. | 27 | N.D. | N.A. | N.A. | 27 | N.A. |
| b-Ala | 1,022 | 970 | 948 | 1,352 | 1,553 | 1,454 | 980 | 38 | 1,453 | 100 |
| DMG | N.D. | N.D. | N.D. | N.D. | N.D. | N.D. | N.A. | N.A. | N.A. | N.A. |
| g-Aminobutyric acid | 212 | 216 | 204 | 521 | 568 | 599 | 211 | 6.3 | 563 | 39 |
| Choline | 277 | 280 | 287 | N.D. | N.D. | N.D. | 282 | 5.4 | N.A. | N.A. |
| Ser | 6,494 | 6,248 | 6,355 | 8,189 | 9,775 | 9,584 | 6,366 | 123 | 9,183 | 866 |
| Carnosine | 5.8 | 0.3 | N.D. | N.D. | 7.4 | 13 | 3.0 | 3.9 | 10 | 4.0 |
| Creatinine | 99 | 118 | 87 | 130 | 164 | 150 | 101 | 15 | 148 | 17 |
| Pro | 7,180 | 6,970 | 7,138 | 10,769 | 13,335 | 12,732 | 7,096 | 111 | 12,278 | 1,342 |
| Val | 1,236 | 1,236 | 1,252 | 2,040 | 2,630 | 2,590 | 1,241 | 9.5 | 2,420 | 330 |
| Betaine | N.D. | N.D. | N.D. | N.D. | N.D. | N.D. | N.A. | N.A. | N.A. | N.A. |
| Thr | 4,443 | 4,394 | 4,672 | 5,453 | 6,574 | 6,380 | 4,503 | 148 | 6,136 | 599 |
| Homoserine | N.D. | N.D. | N.D. | N.D. | N.D. | N.D. | N.A. | N.A. | N.A. | N.A. |
| BTL | N.D. | N.D. | N.D. | N.D. | N.D. | N.D. | N.A. | N.A. | N.A. | N.A. |
| Cys | 54 | 48 | 40 | 145 | 238 | 215 | 47 | 6.9 | 199 | 48 |
| Hydroxyproline | 383 | 381 | 367 | 486 | 548 | 548 | 377 | 8.6 | 528 | 36 |
| Creatine | 14,848 | 14,962 | 15,224 | 13,604 | 15,933 | 16,284 | 15,011 | 193 | 15,273 | 1,456 |
| Leu | 1,078 | 1,094 | 1,131 | 1,713 | 1,983 | 2,078 | 1,101 | 27 | 1,925 | 189 |
| Ile | 1,234 | 1,255 | 1,293 | 1,916 | 2,381 | 2,251 | 1,261 | 29 | 2,183 | 240 |
| Asn | 2,594 | 2,578 | 2,671 | 2,566 | 2,937 | 2,978 | 2,614 | 50 | 2,827 | 227 |
| Ornithine | 160 | 194 | 178 | 320 | 399 | 393 | 178 | 17 | 371 | 44 |
| Asp | 9,236 | 8,749 | 9,276 | 6,930 | 8,161 | 8,419 | 9,087 | 294 | 7,837 | 795 |
| Homocysteine | N.D. | N.D. | N.D. | N.D. | N.D. | N.D. | N.A. | N.A. | N.A. | N.A. |
| Adenine | N.D. | N.D. | N.D. | N.D. | 6.2 | N.D. | N.A. | N.A. | 6.2 | N.A. |
| Hypoxanthine | 35 | N.D. | 43 | 72 | 94 | 96 | 39 | 5.7 | 88 | 14 |
| Spermidine | 65 | 79 | 85 | 100 | 141 | 156 | 77 | 10 | 132 | 29 |
| Gln | 21,086 | 20,400 | 20,946 | 19,152 | 23,976 | 23,674 | 20,810 | 362 | 22,267 | 2,702 |
| Lys | 1,020 | 976 | 996 | 1,320 | 1,661 | 1,645 | 998 | 22 | 1,542 | 192 |
| Glu | 26,723 | 28,184 | 28,599 | 41,853 | 51,044 | 50,036 | 27,835 | 985 | 47,644 | 5,041 |
| Met | 244 | 238 | 229 | 426 | 469 | 462 | 237 | 7.8 | 452 | 23 |
| Guanine | N.D. | N.D. | N.D. | N.D. | N.D. | N.D. | N.A. | N.A. | N.A. | N.A. |
| His | 837 | 884 | 882 | 1,393 | 1,653 | 1,622 | 868 | 27 | 1,556 | 142 |
| Carnitine | 18 | 5.4 | 19 | 69 | 154 | 181 | 14 | 7.6 | 135 | 58 |
| Phe | 668 | 690 | 712 | 1,166 | 1,431 | 1,463 | 690 | 22 | 1,353 | 163 |
| Arg | 1,548 | 1,516 | 1,563 | 2,139 | 2,662 | 2,550 | 1,542 | 24 | 2,450 | 275 |
| Citrulline | 33 | 35 | 38 | 32 | 45 | 46 | 36 | 2.4 | 41 | 7.9 |
| Tyr | 795 | 801 | 831 | 1,319 | 1,572 | 1,534 | 809 | 20 | 1,475 | 136 |
| SAH | N.D. | N.D. | N.D. | N.D. | N.D. | N.D. | N.A. | N.A. | N.A. | N.A. |
| Spermine | N.D. | N.D. | N.D. | N.D. | N.D. | N.D. | N.A. | N.A. | N.A. | N.A. |
| Trp | 234 | 233 | 243 | 345 | 422 | 418 | 237 | 5.4 | 395 | 43 |
| Cystathionine | N.D. | N.D. | N.D. | N.D. | N.D. | N.D. | N.A. | N.A. | N.A. | N.A. |
| Adenosine | N.D. | N.D. | N.D. | N.D. | N.D. | 24 | N.A. | N.A. | 24 | N.A. |
| Inosine | N.D. | N.D. | N.D. | N.D. | 125 | 109 | N.A. | N.A. | 117 | 11 |
| Guanosine | N.D. | N.D. | N.D. | N.D. | N.D. | N.D. | N.A. | N.A. | N.A. | N.A. |
| ArgSuccinate | 70 | 60 | 62 | 93 | 106 | 93 | 64 | 5.7 | 97 | 7.3 |
| GSSG | 507 | 522 | 803 | 909 | 1,031 | 1,203 | 611 | 166 | 1,047 | 148 |
| GSH | 4,901 | 4,352 | 4,800 | 5,913 | 8,272 | 6,791 | 4,684 | 292 | 6,992 | 1,192 |
| SAM | 185 | 178 | 186 | 245 | 279 | 288 | 183 | 3.9 | 270 | 23 |
| No Label | 1.0 | 1.0 | 1.0 | 1.0 | 1.0 | 1.0 | 1.0 | 0.005 | 1.0 | 0.004 |
| No Label | 8,213 | 7,628 | 7,190 | 11,672 | 13,478 | 14,091 | 7,677 | 513 | 13,080 | 1,258 |
| No Label | 1.0 | 1.0 | 1.0 | 1.0 | 1.0 | 1.0 | 1.0 | 0.005 | 1.0 | 0.011 |
| No Label | 1,353 | 1,261 | 1,225 | 1,646 | 1,960 | 2,149 | 1,280 | 66 | 1,918 | 254 |
| No Label | 9.7 | 8.3 | 6.0 | 6.5 | 8.0 | 5.6 | 8.0 | 1.9 | 6.7 | 1.2 |
| No Label | 5,915 | 5,397 | 6,405 | 7,732 | 10,334 | 9,196 | 5,906 | 504 | 9,087 | 1,304 |
| No Label | 1.0 | 1.0 | 1.0 | 1.5 | 0.9 | 1.0 | 1.0 | 0.012 | 1.1 | 0.4 |
| No Label | 0.09 | 0.08 | 0.08 | 0.10 | 0.09 | 0.09 | 0.08 | 0.005 | 0.09 | 0.005 |
| No Label | 12 | 10 | 11 | 30 | 32 | 27 | 11 | 0.6 | 29 | 2.2 |
| No Label | 5.3 | 5.1 | 2.2 | 19 | 23 | 12 | 4.2 | 1.7 | 18 | 5.5 |
| No Label | ##### | ##### | ##### | ##### | ##### | ##### | ##### | 1,537 | ##### | 27,924 |
| No Label | 10,994 | 11,001 | 11,409 | 15,772 | 19,202 | 18,909 | 11,135 | 238 | 17,961 | 1,901 |
| No Label | ##### | ##### | ##### | ##### | ##### | ##### | ##### | 1,303 | ##### | 26,051 |
| No Label | ##### | ##### | ##### | ##### | ##### | ##### | ##### | 1,510 | ##### | 27,570 |
| No Label | 9,472 | 9,443 | 9,878 | 13,232 | 16,023 | 15,769 | 9,598 | 243 | 15,008 | 1,543 |
| No Label | 3,548 | 3,585 | 3,676 | 5,669 | 6,993 | 6,919 | 3,603 | 66 | 6,527 | 744 |
| No Label | 1,697 | 1,724 | 1,786 | 2,830 | 3,425 | 3,415 | 1,736 | 46 | 3,223 | 341 |
| No Label | 2.1 | 2.1 | 2.1 | 2.0 | 2.0 | 2.0 | 2.1 | 0.02 | 2.0 | 0.02 |
| No Label | 57,373 | 57,954 | 59,127 | 75,306 | 92,670 | 90,614 | 58,151 | 893 | 86,197 | 9,487 |
| No Label | 89,327 | 88,746 | 89,672 | ##### | ##### | ##### | 89,248 | 468 | ##### | 16,461 |
| No Label | 3,567 | 3,558 | 3,662 | 5,294 | 6,446 | 6,392 | 3,596 | 58 | 6,044 | 650 |
| No Label | 1,463 | 1,491 | 1,543 | 2,485 | 3,003 | 2,997 | 1,499 | 41 | 2,828 | 297 |
| No Label | 2,714 | 2,730 | 2,774 | 4,382 | 5,479 | 5,302 | 2,739 | 31 | 5,054 | 589 |
| No Label | 11,831 | 11,327 | 11,946 | 9,496 | 11,098 | 11,397 | 11,701 | 330 | 10,664 | 1,022 |
| No Label | 0.15 | 0.12 | 0.12 | 0.3 | 0.3 | 0.3 | 0.13 | 0.02 | 0.3 | 0.014 |
| No Label | 0.2 | 0.2 | 0.2 | 0.10 | 0.11 | 0.12 | 0.2 | 0.02 | 0.11 | 0.009 |
| No Label | 198 | 236 | 248 | 185 | 149 | 157 | 227 | 26 | 164 | 19 |
| No Label | 4.5 | 6.1 | 2.6 | 2.0 | 1.9 | 1.5 | 4.4 | 1.8 | 1.8 | 0.2 |
| No Label | N.A. | N.A. | N.A. | N.A. | N.A. | N.A. | N.A. | N.A. | N.A. | N.A. |
| No Label | 3.5 | 3.3 | 2.9 | 1.9 | 2.1 | 1.9 | 3.2 | 0.3 | 2.0 | 0.11 |

| **Supplementary Table S4: Name of enzymes involved in glycolysis, pentose phosphate pathway and TCA cycle** | |
| --- | --- |
| **Symbol** | **Enzyme** |
| Hk | Hexokinase |
| Pgi | Phosphoglucose isomerase |
| Fbp1 | Fructose 1,6-bisphosphatase |
| Aldo | Aldolase |
| Tpi | Triose phosphate isomerase |
| Gapdh | Glyceraldehyde-3-phosphate dehydrogenase |
| Pgk | Phosphoglycerate kinase |
| Pgm | Phosphoglycerate mutase |
| Eno | Enolase |
| Pk | Pyruvate kinase |
| Ldh | Lactate dehydrogenase |
| Pdk | Pyruvate dehydrogenase kinase |
| Pdh | Pyruvate dehydrogenase |
| G6pd | Glucose-6-phosphate dehydrogenase |
| Pgls | 6-Phosphogluconolactonase |
| Pgd | 6-Phosphogluconate dehydrogenase |
| Rpia | Ribose-5-phosphate isomerase |
| Cs | Citrate synthase |
| Aco | Aconitase |
| Idh2 | Isocitrate dehydrogenase 2 |
| Scs | Succinyl CoA synthetase |
| Sdh | Succinate dehydrogenase |
| Fh | Fumarase |
| Mdh | Malate dehydrogenase |

| **Supplementary Table S5: Characteristics of the Patients (n=66)** |  |
| --- | --- |
| **Variable** | **Number (%)** |
| Age |  |
| <65 | 42(63.6%) |
| ≧65 | 24(36.3%) |
| Sex |  |
| Male | 42(63.6%) |
| Female | 24(36.3%) |
| Embryology |  |
| Primary | 55(83.3%) |
| Recurrrence | 11(16.7%) |
| Pathologic diagnosis# |  |
| Diffuse astrocytoma, IDH-mutant type | 1(1.5%) |
| Diffuse astrocytoma, IDH-Wild type | 2(3.0%) |
| Anaplastic astrocytoma, IDH-mutant type | 3(4.5%) |
| Anaplastic astrocytoma, IDH-wild type | 3(4.5%) |
| Anaplastic astrocytoma, NOS | 1(1.5%) |
| Glioblastoma, IDH-wild type | 26(39.4%) |
| Glioblastoma, IDH-mutant type | 1(1.5%) |
| Glioblastoma, NOS | 6(9.1%) |
| Diffuse midline glioma, H3K27M mutant | 3(4.5%) |
| Oligodendroglioma, IDH-mutant and 1p/19q codeleted | 3(4.5%) |
| Oligodendroglioma, NOS | 4(6.1%) |
| Anaplastic Oligodendroglioma, IDH-mutant and 1p/19q codeleted | 1(1.5%) |
| Anaplastic Oligodendroglioma, NOS | 3(4.5%) |
| Anaplastic Oligoastrocytoma, IDH-mutant type | 1(1.5%) |
| GBM with ologodendroglial component | 6(9.1%) |
| Griomatosis cererbrii | 1(1.5%) |
| High grade glioma | 1(1.5%) |
| GLUT1 |  |
| Positive | 26(39.4%) |
| Negative | 39(59.1%) |
| ## | 1(1.5%) |
| #:WHO 2007 and WHO 2016 |  |
| ##:These sumples were not suitable for immunohistochemistry. The quality was quite poor. | |

| **Supplementary Table 6: Primer for real-time RT-PCR.** | | |
| --- | --- | --- |
| **Symbol** | **Forward(5'-3')** | **Reverse(5'-3')** |
| *H3K27M* | GGCCTATCTGGTTGGCCTTT | CGTCGTCATCCTTGTAATCAGC |
| *Hif-1a* | ATGGGTTATGAGCCGGAAGA | ACCTCTTTTGGCAAGCATCC |
| *Ldha* | TGTCTCCAGCAAAGACTACTGT | GACTGTACTTGACAATGTTGGGA |
| *Glut1 (Slc2a1)* | CAGTTCGGCTATAACACTGGTG | GCCCCCGACAGAGAAGATG |
| *Tpi1* | CCAGGAAGTTCTTCGTTGGGG | CAAAGTCGATGTAAGCGGTGG |
